# A sodium-ion-conducted asymmetric electrolyzer to lower the operation voltage for direct seawater electrolysis

**DOI:** 10.1038/s41467-023-39681-1

**Published:** 2023-07-04

**Authors:** Hao Shi, Tanyuan Wang, Jianyun Liu, Weiwei Chen, Shenzhou Li, Jiashun Liang, Shuxia Liu, Xuan Liu, Zhao Cai, Chao Wang, Dong Su, Yunhui Huang, Lior Elbaz, Qing Li

**Affiliations:** 1 State Key Laboratory of Material Processing and Die & Mould Technology, School of Materials Science and Engineering, Huazhong University of Science and Technology, 430074 Wuhan, Hubei China; 2 Beijing National Laboratory for Condensed Matter Physics, Institute of Physics, Chinese Academy of Sciences, 100190 Beijing, China; 3 Faculty of Materials Science and Chemistry, China University of Geosciences (Wuhan), 430074 Wuhan, Hubei China; 4 School of Materials Science and Engineering, Tongji University, 201804 Shanghai, China; 5 Department of Chemistry and the Institute of Nanotechnology and Advanced Materials, Bar-Ilan University, 5290002 Ramat-Gan, Israel

**Keywords:** Electrocatalysis, Electrocatalysis, Hydrogen energy

## Abstract

Hydrogen produced from neutral seawater electrolysis faces many challenges including high energy consumption, the corrosion/side reactions caused by Cl^-^, and the blockage of active sites by Ca^2+^/Mg^2+^ precipitates. Herein, we design a pH-asymmetric electrolyzer with a Na^+^ exchange membrane for direct seawater electrolysis, which can simultaneously prevent Cl^-^ corrosion and Ca^2+^/Mg^2+^ precipitation and harvest the chemical potentials between the different electrolytes to reduce the required voltage. In-situ Raman spectroscopy and density functional theory calculations reveal that water dissociation can be promoted with a catalyst based on atomically dispersed Pt anchored to Ni-Fe-P nanowires with a reduced energy barrier (by 0.26 eV), thus accelerating the hydrogen evolution kinetics in seawater. Consequently, the asymmetric electrolyzer exhibits current densities of 10 mA cm^−2^ and 100 mA cm^−2^ at voltages of 1.31 V and 1.46 V, respectively. It can also reach 400 mA cm^−2^ at a low voltage of 1.66 V at 80 °C, corresponding to the electricity cost of US$1.36 per kg of H_2_ ($0.031/kW h for the electricity bill), lower than the United States Department of Energy 2025 target (US$1.4 per kg of H_2_).

## Abstract (web summary)

Hydrogen produced directly from neutral seawater is promising but challenging due to seawater’s complex composition. Here, the authors report a Na^+^-conducted pH-asymmetric electrolyzer that can directly split seawater into hydrogen with low electricity cost and nearly zero chloride interference.

## Introduction

The growing need for sustainable energy sources in order to reduce greenhouse gas (GHGs) emissions, and increase energy independence and resilience requires a sustainable and economically viable solution for large energy storage. Hydrogen is considered as the most promising alternative to fossil fuels, owing to its high energy density (142 MJ Kg^−1^) and emissions-free use^1,2^. Water electrolysis is an efficient and sustainable route to produce high-purity H_2_, which can make full use of sustainable, yet intermittent energy sources supplied by wind, solar, etc.^3^. However, the large-scale application of electrolyzers using high-purity deionized water may be limited^4^. Seawater is the most significant natural resource which accounts for 96.5% of the total water on earth, but it is still underutilized for water electrolysis^5,6^. Moreover, direct seawater electrolysis can take full advantage of the seaside wind energy, with a potential of producing more than 13,500 terawatt hours per year in the United States alone, approximately 3 times its annual electricity consumption^7,8^, which makes seawater electrolysis a very appealing approach for green H_2_ production.

However, natural seawater is a complex neutral electrolyte (pH ~ 8), containing multiple ions, among which the main constituents are Cl^−^ and Na^+^^5^. The major issues associated with direct seawater electrolysis are: (1) the undesired anodic chloride oxidation reactions (ClOR), i.e., the chlorine evolution (2Cl^−^ → Cl_2_ + 2e^−^) and the formation of hypochlorite (Cl^−^ + 2OH^−^ → ClO^−^ + H_2_O + 2e^−^), competing with the oxygen evolution reaction (OER)^9^; (2) the high energy cost caused by the sluggish reaction kinetics for hydrogen evolution reaction (HER) and OER in the neutral medium without a buffer^10^; and (3) other ions such as Ca^2+^ and Mg^2+^ that may precipitate and block the catalytic active sites^11^. These issues pose great challenges to the design of electrolyzers and catalysts for efficient direct seawater electrolysis. Most of the research has been focused on alkaline seawater electrolysis, for which alkali is added to the seawater to suppress the evolution of chlorine^12–14^. However, chloride still remain in these systems to some extent, and they generally suffer from inferior activity compared with alkaline electrolyzers working with deionized water^15^. More recently, an asymmetric electrolyte feed method with an anion exchange membrane as the separator has been reported for seawater electrolysis^9^, but Cl^-^ still seem to cross the membrane to the anode under the electric field, which not only lead to undesirable ClOR at the anode but also destroy the membrane. Moreover, the asymmetric feed electrolyzer demonstrates an inferior performance to that of symmetric KOH feed due to the sluggish kinetics of HER in natural seawater without buffer media. Therefore, it is highly desirable to develop an electrolysis system with efficient and robust catalysts that will inhibit ClOR and realize direct seawater electrolysis under low voltage^16^.

Herein, we present our newly developed pH-asymmetric feed electrolyzer using a Na^+^ exchange membrane as a separator for efficient and energy-saving direct seawater electrolysis for the first time. The Na^+^ exchange membrane could prevent the transport of Cl^-^ ions in the catholyte (NaCl solution or natural seawater) to the anode (NaOH), thus avoiding the competing ClOR. The problem of Ca^2+^ and Mg^2+^ precipitates will be alleviated for the HER in the near-neutral seawater (pH <9.5) and flow electrolyte. The energy cost of the electrolyzer can also be significantly reduced due to the pH-asymmetric framework as well as the accelerated reaction kinetics benefited from rationally designed amorphous Ni_6.6_Fe_0.4_P_3_ NWs supported Pt single atoms (denoted as Pt_SA_-Ni_6.6_Fe_0.4_P_3_) and amorphous Ni_5_Fe_2_P_3_ NWs as efficient anode and cathode catalysts, respectively. As a result, the asymmetric electrolyzer displays the current densities of 10 mA cm^−2^ and 100 mA cm^−2^ at voltages of 1.31 V and 1.46 V, respectively, outperforming the state-of-the-art electrolyzers for seawater electrolysis^15,17–27^. In addition, it can reach a current density of 400 mA cm^−2^ at a low voltage of 1.66 V at 80 °C (iR-compensation-free), corresponding to an electricity cost of US$1.36 per kg of H_2_ (3.96 kWh per m^3^ H_2_, $0.031/kW h for the electricity bill), which is lower than the U.S. Department of Energy (DOE) 2025 target of US$1.4 per kg of H_2_^28^. The accelerated water dissociation process as well as the superhydrophilic surface for Pt_SA_-Ni_6.6_Fe_0.4_P_3_ are suggested to account for the enhanced catalytic performance.

## Results

### Electrolyzer design and electrocatalyst characterizations

**Fig. 1:**
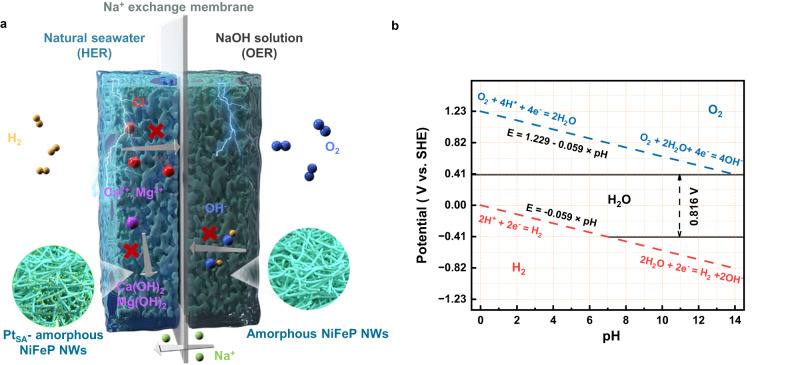
Design strategies of pH-asymmetric electrolyzer. **a** Scheme for the asymmetric electrolyzer with sodium ions exchange membrane. **b** The Pourbaix diagram of water.

The scheme of the electrolyzer with an asymmetric electrolyte feed developed in this work is shown in Fig. 1a. Bipolar chambers are separated by treated Na^+^ exchange membrane (details in the Methods section), which prevents the Cl^-^ transportation through the membrane to the anode, thus avoiding the undesired ClOR^29^. Both the anode and cathode catalysts are supported on porous Ni electrodes which also serves as the flow channels at the same time. In the asymmetric electrolyzer, NaCl solution, or natural seawater is circulated in the cathode chamber, while NaOH solution is circulated in the anode chamber. Since both half reactions in water splitting are pH-sensitive redox reactions, as manifested in the Pourbaix diagram of water (Fig. 1b), the chemical potential difference between electrolytes of different pH in cathode and anode can be used to reduce the required voltage (from 1.23 V to 0.816 V) for direct seawater electrolysis^30^. The dependence of the electrolyzer half reactions and corresponding electrode potentials as function of the electrolyte pH are given in Supplementary Note 1. The issue with Mg^2+^ and Ca^2+^ precipitates in seawater electrolyzer could be alleviated in the asymmetric electrolyzer with Na^+^ ions exchange membrane, since these hydroxide precipitates are usually formed at pH >9.5 and greatly influenced by the electrolyte flow rate^31^.

**Fig. 2:**
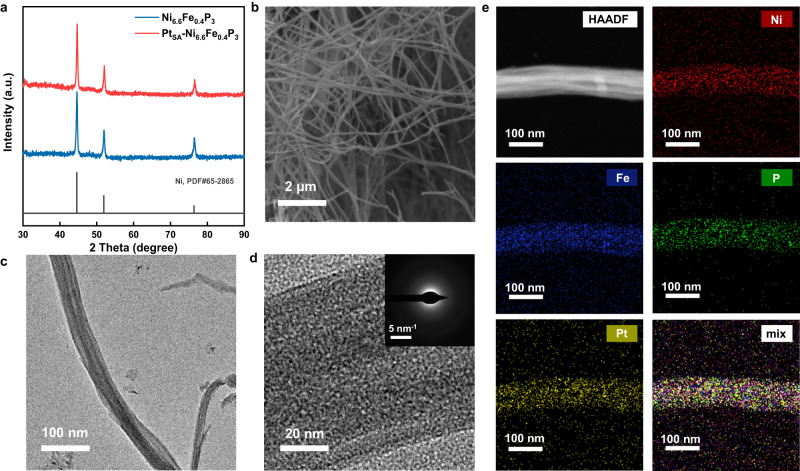
The morphology and structure characterization of the fabricated Pt_SA_-Ni_6.6_Fe_0.4_P_3_ catalyst. **a** XRD patterns of Pt_SA_-Ni_6.6_Fe_0.4_P_3_ and Ni_6.6_Fe_0.4_P_3_. **b** SEM image of Pt_SA_-Ni_6.6_Fe_0.4_P_3_ NWs. **c** TEM image of Pt_SA_-Ni_6.6_Fe_0.4_P_3_ NWs. **d** HRTEM image of Pt_SA_-Ni_6.6_Fe_0.4_P_3_ (inset shows the corresponding FFT). **e** HAADF-STEM image and the corresponding EDS elemental mapping analysis of Pt_SA_-Ni_6.6_Fe_0.4_P_3_.

In order to lower the energy consumption in direct seawater electrolysis, efficient catalysts should be rationally designed. Herein, we display a simple method to synthesize amorphous Ni-Fe-P NWs and single-atom Pt loaded amorphous Ni-Fe-P NWs as OER and HER catalysts, respectively. In brief, we used an electrodeposition method to form hierarchically porous Ni-Fe alloy on Ni foam (Supplementary Fig. 1), followed by a solvothermal method^13^ where the Ni-Fe alloy reacted with red phosphorus in diethylene glycol to form Ni-Fe-P NWs. Ni_x_Fe_1-x_P_3_ with varying ratios of Ni to Fe can be easily synthesized by adjusting the ratio of Ni^2+^ and Fe^2+^ in the electrolyte during electrodeposition (for details, see Methods section). The actual elemental compositions of these catalysts were investigated using X-ray Fluorescence Spectrometry (XRF) and the results are summarized in Supplementary Table 1. Scanning electron microscopy (SEM; Supplementary Fig. 2) images show that interwoven Ni_x_Fe_1-x_P_3_ NWs were in situ formed inside the Ni foam after phosphatizing. X-ray diffraction (XRD) patterns show that only the peaks of Ni foam can be detected, which indicate the amorphous nature of Ni_x_Fe_1-x_P_3_ NWs (Supplementary Fig. 3). Pt_SA_-Ni_6.6_Fe_0.4_P_3_ catalyst was obtained by electroreduction process with cyclic voltammetry in 1 M NaOH containing ultralow Pt salts concentration, and single atoms of Pt (1.35 wt%, measured using inductively coupled plasma-mass spectrometry) were anchored on the defect-rich amorphous Ni_6.6_Fe_0.4_P_3_ NWs due to its disordered long-range atomic arrangement^32^. The XRD pattern of Pt_SA_-Ni_6.6_Fe_0.4_P_3_ on Ni foam is shown in Fig. 2a, in which no Pt characteristic peaks were detected, implying the absence of crystalline Pt. In addition, the XRD pattern (Supplementary Fig. 4) of the powder that was scraped from the surface of the Pt_SA_-Ni_6.6_Fe_0.4_P_3_ displays no obvious peaks, which further proves the amorphous feature of Pt_SA_-Ni_6.6_Fe_0.4_P_3_. SEM and transmission electron microscopy (TEM) images also support that Pt_SA_-Ni_6.6_Fe_0.4_P_3_ form an interwoven NWs framework with a NW diameter of about 60 nm (Fig. 2b, c). Moreover, no lattice fringes can be observed on the Pt_SA_-Ni_6.6_Fe_0.4_P_3_ NW (Fig. 2d). The fast Fourier transform (FFT) image (inset of Fig. 2d) of Pt_SA_-Ni_6.6_Fe_0.4_P_3_ also displays a diffuse center spot, indicating its maintaining of amorphous phase, which is consistent with the result of XRD patterns. In addition, elemental mappings of Pt_SA_-Ni_6.6_Fe_0.4_P_3_ confirm the presences and uniform distributions of Pt, Ni, Fe and P on the NW (Fig. 2e). The high-angle annular darkfield STEM (HAADF-STEM, Supplementary Fig. 5) was used to investigate the distribution of Pt over Ni_6.6_Fe_0.4_P_3_. The bright spots appear on the amorphous structure, corresponding to heavy constituent atom species, which clearly confirms the immobilization of atomically dispersed Pt atoms.

**Fig. 3:**
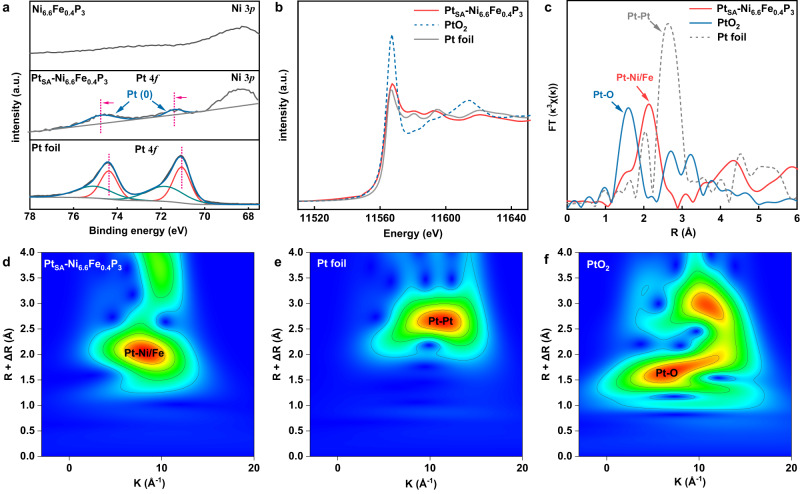
Fine structure characterizations of Pt_SA_-Ni_6.6_Fe_0.4_P_3_ catalyst. **a** XPS spectra of Pt 4 *f* for Pt_SA_-Ni_6.6_Fe_0.4_P_3_ and Pt foil. **b** Pt L3-edge XANES spectra of Pt_SA_-Ni_6.6_Fe_0.4_P_3_, PtO_2_ and Pt foil. **c** Corresponding Pt L3 **k**^3^-weighted FT-EXAFS curves of Fig. 3b. **d**–**f** EXAFS wavelet transform plots of Pt_SA_-Ni_6.6_Fe_0.4_P_3_, PtO_2_, and Pt foil.

X-ray photoelectron spectroscopy (XPS) was used to study the surface element states of the catalyst. As shown in Fig. 3a, the peaks of Pt 4 *f* for Pt_SA_-Ni_6.6_Fe_0.4_P_3_ shift positively (~0.3 eV) compared to the Pt^0^ peaks for Pt foil, indicating the electrons are transformed from Pt to the Ni_6.6_Fe_0.4_P_3_ support. Notably, the peak at 68.3 eV is attributed to Ni 3*p*, which can also be detected for Ni_6.6_Fe_0.4_P_3_ support^33^. The XPS spectra of Ni and P in the Pt_SA_-Ni_6.6_Fe_0.4_P_3_ before and after the addition of Pt are shown in Supplementary Fig. 6, in which the peaks of P 2*p* show a negative shift, further demonstrating the electronic interaction between Pt and the support. X-ray absorption near-edge spectroscopy (XANES) of Pt_SA_-Ni_6.6_Fe_0.4_P_3_, standard Pt foil, and PtO_2_ are shown in Fig. 3b. the white-line intensity has been reported to correspond to the transfer of the Pt 2*p*_3/2_ core-electron to 5*d* states, and thus is used as an indicator for Pt 5*d*-band occupancy^34^. The slightly higher white-line intensity of Pt_SA_-Ni_6.6_Fe_0.4_P_3_ than that of Pt foil indicates the more unoccupied 5*d* orbitals for single-atom Pt, which further certifies the charge loss of the single-atom Pt after coordinating with the supports, in line with the XPS analysis in Fig. 3a. To capture the atomic coordination information of Pt, the extend X-ray absorption fine structure (EXAFS) analysis was carried out on the Pt_SA_-Ni_6.6_Fe_0.4_P_3_ catalyst. Figure 3c shows the **k**^3^-weighted Fourier transform (FT) curves at R space of Pt L3-edge EXAFS spectra for Pt_SA_-Ni_6.6_Fe_0.4_P_3_ in comparison with the references of PtO_2_ and Pt foil. It shows that the peak of typical Pt-Pt bond for Pt foil at about 2.7 Å is absent in the Pt_SA_-Ni_6.6_Fe_0.4_P_3_, which strengthens the postulate that the Pt is atomically dispersed and does not form nano-particles or small clusters. The peak at 2.1 Å for Pt_SA_-Ni_6.6_Fe_0.4_P_3_ is associated with the Pt-Ni/Fe bonds, in which the Pt-Ni and Pt-Fe bonds are difficult to distinguish due to the similar bond lengths^34,35^. In addition, the wavelet transform analysis (Fig. 3d–f) was carried out to further prove the single-atom Pt dispersion on Ni_6.6_Fe_0.4_P_3_^36,37^. The intensity maximum at 11 Å^−1^ which corresponds to Pt-Pt is absent for Pt_SA_-Ni_6.6_Fe_0.4_P_3_, which also confirms that the Pt atoms are atomically dispersed on the catalysts. A main Pt-Ni coordination is taken to simplify the modeling of this system in EXAFS fitting due to the relatively large Ni:Fe ratio (33:2). The first-shell EXAFs fitting of Pt_SA_-Ni_6.6_Fe_0.4_P_3_ gives a coordination number of 1.0 for Pt-O contribution and 3.3 for Pt-Ni contribution (Supplementary Fig. 7 and Supplementary Table 2), suggesting that the Pt atoms are mainly anchored at the hollow sites between Ni atoms.

### Electrocatalytic performance for seawater electrolysis

**Fig. 4:**
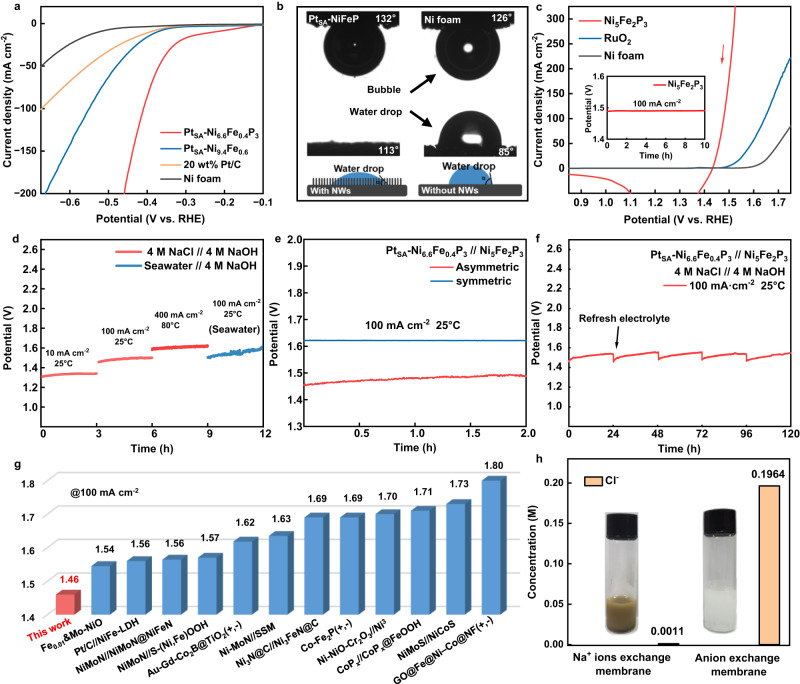
Electrochemical performance. **a** HER polarization curves of Pt_SA_-Ni_6.6_Fe_0.4_P_3_, Pt_SA_-Ni-Fe, Pt/C and Ni foam in 4 M NaCl with 90% iR-compensation. (resistance value: 1.1 ± 0.1 Ω) **b** Air-bubble contact angles under water (top), staticwater-droplet contact angles (middle) and the structure Schematic illustration (bottom, Wenzel model) for Pt_SA_-Ni_6.6_Fe_0.4_P_3_ and Ni foam. **c** OER polarization curves of Ni_5_Fe_2_P_3_, RuO_2_ and Ni foam in NaOH and chronopotentiometry curve (inset) with 90% iR-compensation. (resistance value: 1.3 ± 0.1 Ω) **d** Chronopotentiometry curves of asymmetric electrolyzers at different conditions (catalyst size: 0.5 × 1 cm^2^, Pt loading: 40 μg). **e** Chronopotentiometry curve of asymmetric and symmetric electrolyzers at 100 mA cm^−2^. **f** Long-term stability test of the asymmetric electrolyzer at constant 100 mA cm^−2^. **g** Operation voltage of the state-of-the-art seawater electrolyzers for overall seawater electrolysis at 100 mA cm^−2^. **h** Concentration of Cl^-^ ions of anode electrolytes for asymmetric electrolyzers with Na^+^/anion exchange membrane and the photographs after adding AgNO_3_ solution (inset).

The HER performance was evaluated first with the as-prepared catalysts in 4 M NaCl solution. For comparison, the Ni-Fe alloy supports Pt single atoms (denoted as Pt_SA_-Ni-Fe, Methods), Pt/C (20 wt%), Ni foam and Ni_6.6_Fe_0.4_P_3_ were also studied under the same conditions (Fig. 4a and Supplementary Fig. 8). The Pt_SA_-Ni_6.6_Fe_0.4_P_3_ exhibits the highest HER activity among all of the above-mentioned catalysts, and requires an overpotential of 408 mV for a HER current density of 100 mA cm^−2^. The overpotentials required to achieve the same current density with Pt_SA_-Ni-Fe (551 mV) and Pt/C (673 mV) are much higher, which hints on the contribution of the amorphous Ni_6.6_Fe_0.4_P_3_ substrate to the HER electrocatalytic activity of the atomically dispersed Pt sites. In addition, the Pt_SA_-Ni_6.6_Fe_0.4_P_3_ exhibits a smaller Tafel slope of 131 mV dec^−1^ than that of Pt_SA_-Ni-Fe and commercial Pt/C catalysts as shown in Supplementary Fig. 9, which indicates the faster HER kinetic of Pt_SA_-Ni_6.6_Fe_0.4_P_3_^38^. The HER activity of Pt_SA_-Ni_x_Fe_1-x_P_3_ catalysts with different Ni:Fe ratios were also studied here, and the results are presented in Supplementary Fig. 10. Among these catalysts, the Pt_SA_-Ni_6.6_Fe_0.4_P_3_ with the Ni:Fe ratio of 6.6:0.4 shows the best HER performance. In addition, the electrochemical active surface area (ECSA) was calculated for different supports by measuring the double-layer capacitance (*C*_dl_) from their CVs (Supplementary Fig. 11). The *C*_dl_ of Ni_6.6_Fe_0.4_P_3_ is 17.30 mF cm^−2^, relatively high, owing to its NW morphology, and is 2.4 times higher than that of Ni-Fe alloy (7.09 mF cm^−2^), demonstrating the highly improved ECSA and the possibly increased number of active sites after P doping. Interestingly, the Ni_x_Fe_1-x_P_3_ catalysts with different Ni:Fe ratios exhibit similar ECSA, indicating that the intrinsic activity of the active sites for the catalysts are different. The HER performance of the different catalysts measured in 4 M NaCl and normalized by ESCA are presented in Supplementary Fig. 12. There, the ECSA-normalized performance of the Pt_SA_-Ni_6.6_Fe_0.4_P_3_ is the highest of all in terms of current density, indicating its high intrinsic HER activity. Furthermore, the mass activity of Pt_SA_-Ni_6.6_Fe_0.4_P_3_ measured at an overpotential of 400 mV and normalized to the Pt loading is 2.17 A mg^−1^, about 12 times greater than that of commercial Pt/C (0.18 A mg^−1^) (Supplementary Fig. 13), emphasizing that the atomically dispersed Pt supported on Ni_6.6_Fe_0.4_P_3_ could promote HER in neutral media better than commercial Pt/C. The HER activity of Pt_SA_-Ni_6.6_Fe_0.4_P_3_ in 1 M NaCl and natural seawater are also investigated (Supplementary Fig. 14). After 90% iR-compensation, the Pt_SA_-Ni_6.6_Fe_0.4_P_3_ catalyst reveals the similar HER activity in 4 M NaCl, 1 M NaCl and natural seawater. To better illustrate the stability of the Pt_SA_-Ni_6.6_Fe_0.4_P_3_ catalyst in natural seawater, the HER stability test in natural seawater for Pt_SA_-Ni_6.6_Fe_0.4_P_3_ with a large container is investigated (Supplementary Fig. 15). There is almost no performance degradation with the stirring of the solution at 100 mA cm^−2^ for 18 h, indicating the durability of Pt_SA_-Ni_6.6_Fe_0.4_P_3_. Moreover, Pt_SA_-Ni_6.6_Fe_0.4_P_3_ demonstrates excellent HER performance in 1 M NaOH and 1 M PBS (phosphate-buffered saline, pH = 7, Supplementary Figs. 16 and 17). The Pt_SA_-Ni_6.6_Fe_0.4_P_3_ shows low overpotentials of 89 mV and 240 mV required to reach 100 mA cm^−2^ in 1 M NaOH and 1 M PBS, respectively, which is one of the best reported HER catalysts^34,39^.

Since the HER performance at high current densities is greatly influenced by the wettability of the electrode surface^40^, contact-angle measurements were performed with Pt_SA_-Ni_6.6_Fe_0.4_P_3_ electrode. As shown in Fig. 4b, Pt_SA_-Ni_6.6_Fe_0.4_P_3_ is superhydrophilic, as the droplets spread immediately once in contact with the surface (details in Supplementary Fig. 18). The underwater gas-bubble contact-angles were measured as 132° and 126° for Pt_SA_-Ni_6.6_Fe_0.4_P_3_ and Ni foam, demonstrating the lower adhesive force to the bubbles for Pt_SA_-Ni_6.6_Fe_0.4_P_3_^41^. The smaller and denser bubbles during HER (Supplementary Fig. 19) for Pt_SA_-Ni_6.6_Fe_0.4_P_3_ also confirms this observation. The superhydrophilic Pt_SA_-Ni_6.6_Fe_0.4_P_3_ with lower adhesive force to the bubbles results in fast bubble release from the electrode surface, thus exposing more active sites and improving its HER performance in NaCl solution^41^.

The Ni_x_Fe_1-x_P_3_ support also exhibits excellent OER activity in 1 M NaOH. The OER polarization curve was collected by the negative sweeping to exclude the interference of Ni^2+^ oxidation current with the actual OER current (Supplementary Fig. 20)^42^. As shown in Fig. 4c and Supplementary Fig. 21, the Ni_5_Fe_2_P_3_ shows the highest OER performance among all the catalysts with a quite low overpotential of 248 mV to reach the current density of 100 mA cm^−2^. More importantly, the catalyst shows no degradation over a period of 10 h continuous operation (inset in Fig. 4c), confirming its excellent stability in half-cell. Tafel plots constructed from this data show that the Ni_5_Fe_2_P_3_ has a relatively smaller slope of 30 mV dec^−1^ in comparison with that of the RuO_2_ (63 mV dec^−1^) and Ni foam (81 mV dec^−1^), verifying its rapid OER catalytic kinetics (Supplementary Fig. 22)^43^.

The rotating ring-disk electrode technique was used to quantitatively detect the local pH on the Pt_SA_-Ni_6.6_Fe_0.4_P_3_ for HER in NaCl solution (Supplementary Note 2 and Supplementary Figs. 23 and 24)^44^. We find that the pH value on the Pt_SA_-Ni_6.6_Fe_0.4_P_3_ increases when HER occurs but it is still far lower than the anode pH (14.4), indicating that the chemical potential due to the pH difference can be leveraged to increase the efficiency of the process. The developed catalysts were then applied for the designed asymmetric electrolyzer with Pt_SA_-Ni_6.6_Fe_0.4_P_3_ and Ni_5_Fe_2_P_3_ as cathode and anode catalysts, respectively. The cathode chamber was circuited with 4 M NaCl solution and anode chamber was circuited with 4 M NaOH solution to maintain the balance of the concentration for Na^+^ between two half cells. The electrolyzer assembly is presented in Supplementary Fig. 25. Remarkably, the asymmetric electrolyzer reaches the current density of 10 mA cm^−2^ at a low voltage of 1.31 V, and a current density of 100 mA cm^−2^ at 1.46 V and 1.50 V for 4 M NaCl solution and natural seawater as cathode electrolyte, respectively (Fig. 4d), which outperform the state-of-the-art electrolyzers operating with seawater. Meanwhile, the symmetric electrolyzer with the same catalysts requires a voltage of 1.62 V to reach a current density of 100 mA cm^−2^ with 4 M NaOH as the electrolyte (Fig. 4e), indicating that the asymmetric electrolyzer with the above catalysts could significantly reduce the energy cost of water electrolysis. The long-term stability performance of the electrolyzer are further shown in Fig. 4f and Supplementary Fig. 26. Slight voltage increases were observed, which may due to increase pH of the NaCl solution during long-term test, in which the proton will be consumed if the solution is cycled. The pH of anode and cathode electrolyte of the asymmetric electrolyzer over time at 100 mA cm^−2^ was also monitored over 12 h when the electrolytes were cycled. As shown in Supplementary Fig. 27, the pH of cathode electrolyte increases from 6.9 to 8.8, and the pH of anode electrolyte slightly drops from 14.4 to 14.1, which may be responsible for the performance degradation of the asymmetric electrolyzer for water electrolysis. However, the pH of the output cathode electrolyte (Supplementary Fig. 28) could be maintained at about 8.5 when operating at 100 mA cm^−2^ in one-way flow scheme, and the electrolyte with stable pH can exhibit stable voltage response. After 120 h water splitting test, the voltage required to reach a current density of 100 mA cm^−2^ shows only very little increase after refreshing the solution, verifying the superior durability of the system. When using the asymmetric electrolyzer working under industrial operating conditions of 400 mA cm^−2^ and 80 °C for water splitting with one-way flow scheme, the operating voltage is only 1.66 V without iR-compensation (Supplementary Fig. 29), which is also significantly lower than the symmetric electrolyzer (1.83 V, Supplementary Fig. 30). The stable voltage proves the stability for the asymmetric electrolyzer with one-way flowing feed scheme to stabilize the pH on the cathode. The electricity cost of the asymmetric electrolyzer is reduced to 3.96 kWh per m^3^ H_2_, corresponding to US$1.36 per kg of H_2_, which is lower than the U.S. DOE 2025 target of US$1.4 per kg of H_2_ and may be further reduced through engineering system architecture. The total hydrogen levelized cost is estimated at US$1.96 per kg of H_2_, slightly lower than U.S. DOE 2025 target of US$2 per kg of H_2_^28^. In addition, according to the calculation framework proposed by Enapter^45^ (which takes full account of the operating and maintenance costs over the lifetime if the electrolyzer is used on a large-scale), the total hydrogen levelized cost of our electrolyzer is estimated to be €3.79 per kg of H_2_, which is much lower than the projected price for the mass-produced EL Model T in 2023/2024 of €4.15 per kg of H_2_ (details in Supplementary Note 3). The electrolyzer could further reach a higher current density (500 mA cm^−2^, 65 °C) at the voltage of 1.72 V, indicating the high performance of the asymmetric electrolyzer for water electrolysis (Supplementary Fig. 31). Figure 4g summarizes the comparation of the operating voltage of the state-of-the-art seawater electrolyzers for overall seawater electrolysis at 100 mA cm^−2^ and 25 °C^15,17–27^. In addition, our electrolyzer requires an about 25.3% less in electrical energy consumption at 400 mA cm^−2^ compared to the reported direct seawater electrolysis, which combines seawater purification and subsequent electrolysis^46^. It is obvious that our sodium ions conducted asymmetric electrolyzer with Pt_SA_-Ni_6.6_Fe_0.4_P_3_ and Ni_5_Fe_2_P_3_ as the catalysts achieves the lowest energy consumption. To further verify the stability of the asymmetric electrolyzer for direct seawater electrolysis, we applied turbid sea salt water containing Ca^2+^ and Mg^2+^ in the cathode chamber with the one-way flowing feed scheme to maintain the electrolyte pH (Supplementary Fig. 32). The electrolyzer demonstrates relatively stable voltage response at 100 mA cm^−2^. Moreover, white precipitates are not observed on both the electrodes and the membrane after 14 h. These phenomena further certify the stability of the electrolyzer and the catalysts therein.

The changes in structure and catalytic performance of Pt_SA_-Ni_6.6_Fe_0.4_P_3_ and Ni_5_Fe_2_P_3_ after the stability test were also studied (Supplementary Figs. 33–35). The XRD of Pt_SA_-Ni_6.6_Fe_0.4_P_3_ after stability test shows no typical peaks for Mg(OH)_2_ and Ca(OH)_2_, indicating almost no Ca^2+^ or Mg^2+^ precipitates forms under the effect of flow seawater during continuous operation. Moreover, they both demonstrate negligible morphology change and activity degradation, further indicating their excellent stability. In addition, the elemental composition of the catalysts after 24 h stability test has not significantly changed (Supplementary Table 3). The mass ratio of Pt in Pt_SA_-Ni_6.6_Fe_0.4_P_3_ is nearly unchanged (1.32 vs. 1.35 wt%), confirming the high stability of the catalysts. The anode electrolyte was also analyzed to prove that the Na^+^ exchange membrane can prevent the transport of Cl^-^ to the anode. Ion chromatography was used to detected specific concentration of Cl^-^ in the anode electrolyte with different membranes (Fig. 4h and Supplementary Fig. 36). The concentration of Cl^-^ of the anode electrolyte with Na^+^ exchange membrane is only 0.0011 M, while that with anion exchange membrane is 0.1964 M, which proves the ability of the Na+ exchange membrane to prevent Cl^-^ crossover. As shown in the inset of Fig. 4h (details in Supplementary Fig. 37), large amount of white AgCl precipitate appeared in the anode electrolyte after 12 h electrolysis for the electrolyzer with commercial anion exchange membrane when adding AgNO_3_, while brown precipitate that corresponding to Ag_2_O appears in the anode electrolyte for asymmetric electrolyzer with Na^+^ exchange membrane, further indicating that almost no Cl^-^ ions crossed the Na^+^ exchange membrane to the anode. Therefore, side reactions such as ClOR and the potential corrosion of Cl^-^ on the anode^5^ can be diminished.

### Mechanism study

**Fig. 5:**
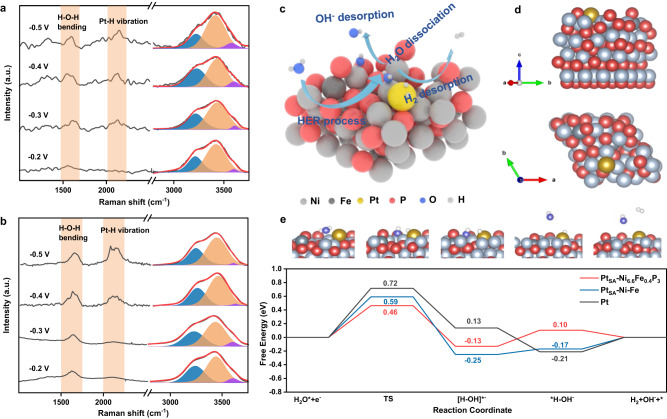
Mechanism study. **a** The in situ Raman spectroscopy in NaCl solution at the surface of Pt_SA_-Ni_6.6_Fe_0.4_P_3_. **b** The in situ Raman spectroscopy in NaCl solution at the surface of Pt foil. **c** Mechanism of Pt_SA_-Ni_6.6_Fe_0.4_P_3_ for HER in NaCl solution. **d** Computational model of Pt_SA_-Ni_6.6_Fe_0.4_P_3_. **e** Free energy diagram of HER in NaCl solution on these three catalyst surfaces. TS represents the activated H_2_O dissociation energy of the transition state. Top: Structural configurations of various states on the Pt_SA_-Ni_6.6_Fe_0.4_P_3_ surface during the HER reaction. (gray: nickel, dark gray: iron, red: phosphorus, yellow: platinum, blue: oxygen, white: hydrogen).

Our previous work has proved that amorphous Ni-Fe-P is an efficient OER catalyst due to the incorporation of phosphorus atoms that breaks the scaling relations of adsorbed OER intermediates and facilitates the OER kinetics^13^. To further understand the OER performance of amorphous Ni_5_Fe_2_P_3_ NWs in this work, in situ Raman spectroscopy of Ni_5_Fe_2_P_3_ was investigated at OER-relevant potential region (1.2 to 1.5 V_RHE_) (Supplementary Fig. 38 and Supplementary Note 4). The peaks observed at 1.4 V_RHE_ can be attributed to NiOOH, indicating that the surface reconstruction of amorphous Ni_5_Fe_2_P_3_ to metal hydroxide during OER test. The hydroxide with remained P would contribute to the excellent OER performance of Ni_5_Fe_2_P_3_^13^. The in situ Raman spectroscopy of Pt_SA_-Ni_6.6_Fe_0.4_P_3_ at the potential window of −0.5 to −0.2 V_RHE_ in NaCl solution, where HER is expected to take place, was also carried out to elucidate the mechanism for the catalyst enhanced HER activity. The extensive band from 3000 cm^−1^ to 3800 cm^−1^ in Fig. 5a, b is ascribed to the O-H stretching mode of interfacial water (*ν*_O-H_), which is sensitive to the chemical environment^47^. The *ν*_O-H_ band can be separated into three Gaussian peaks as suggested, corresponding to three types of O-H stretching vibrations^48^. The low wavenumber component (Fig. 5a, b, blue) and middle component (Fig. 5a, b, orange) are ascribed to 4-coordinated hydrogen-bonded water and 2-coordinated hydrogen-bonded water, respectively. The high wavenumber component (Fig. 5a, b, purple) is attributed to the Na^+^ hydrated water with weak hydrogen-bonded interaction^49^. The peak area of Na^+^ hydrated water increases as the potential decreases for Pt_SA_-Ni_6.6_Fe_0.4_P_3_ compared to Pt foil, indicating the interfacial water structure could be reoriented to more beneficial conditions for water dissociation at negative potentials for Pt_SA_-Ni_6.6_Fe_0.4_P_3_, which may be ascribed to the effect of P as the electron acceptor^50,51^. The first step of HER in unbuffered neutral media is generally considered to be a slow kinetic water molecule dissociation process^52^. Therefore, the promoted water dissociation process on Pt_SA_-Ni_6.6_Fe_0.4_P_3_ would contribute to its improved HER performance. In addition, the band at 1600 cm^−1^ is ascribed to the H-O-H bending mode (*δ*_H-O-H_) of the interfacial water, and the band at 2030 cm^−1^ to 2100 cm^−1^ is attribute to the vibration mode of H atoms coordinated on the top of the surface Pt atom (*ν*_Pt-H_)^53^. As shown in Fig. 5a, b, the band of *δ*_H-O-H_ exists at −0.2 V_RHE_ while the band of *ν*_Pt-H_ is absent for both Pt_SA_-Ni_6.6_Fe_0.4_P_3_ and Pt foil, indicating that the first adsorption layer are water molecules at this potential. However, the band of *ν*_Pt-H_ appears at −0.3 V_RHE_ for Pt_SA_-Ni_6.6_Fe_0.4_P_3_ catalyst, which is more positive than that of the Pt (−0.4 V_RHE_), further revealing that amorphous Pt_SA_-Ni_6.6_Fe_0.4_P_3_ may benefit the dissociation of the adsorbed water and exhibit enhanced HER activity.

Based on the analysis of in situ Raman spectroscopy and EXAFS, we propose that water molecules are first adsorbed at the Ni sites adjacent to Pt atoms, followed by their dissociation and the formation of adsorbed H at the Pt sites. Finally, the adsorbed H atoms at Pt sites are released and H_2_ is formed (Fig. 5c). Density functional theory (DFT) calculations were further investigated by an implicit solvation calculation to disclose the enhanced HER kinetics on the amorphous Ni_6.6_Fe_0.4_P_3_ supported Pt single atoms in NaCl solution which can consider the solvent effect of the solvents and ion solvation^54^. The models of Pt_SA_-Ni_6.6_Fe_0.4_P_3_, Pt_SA_-Ni-Fe and Pt foil were built based on the structural characterization in previous section (Fig. 5d and Supplementary Fig. 39, details in Supplementary Note 5). The energy barrier of HER in NaCl on various catalysts includes two key steps: the water dissociation and H* desorption (Supplementary Fig. 40 and Supplementary Note 6)^32^. As shown in Fig. 5e, the energy barrier of water dissociation, which is the rate-determining step on Pt_SA_-Ni_6.6_Fe_0.4_P_3_ (0.46 eV), is lower than that of Pt foil (0.72 eV). It indicates that Pt_SA_-Ni_6.6_Fe_0.4_P_3_ could facilitate the water dissociation, which is consistent with the in situ Raman spectroscopy. The Pt_SA_-Ni-Fe has a higher energy barrier of water dissociation (0.59 eV) compared to Pt_SA_-Ni_6.6_Fe_0.4_P_3_, indicating that the amorphous Ni_6.6_Fe_0.4_P_3_ formed after P-doping is the key structure to promote the water dissociation process. Moreover, the free energy of desorption of H* on Pt_SA_-Ni_6.6_Fe_0.4_P_3_ (0.10 eV) is closer to zero compared with that on Pt_SA_-Ni-Fe (−0.17 eV) and Pt foil (−0.21 eV), indicating a faster desorption of H*. The lower energy barrier of water dissociation and a faster desorption of H* account for the best HER performance of Pt_SA_-Ni_6.6_Fe_0.4_P_3_.

## Discussion

In summary, we demonstrate a pH-asymmetric electrolyzer using a Na^+^ exchange membrane as the separator and amorphous Ni-Fe-P NWs as the main catalysts to realize direct seawater electrolysis under low voltage. The Na^+^ exchange membrane can prevent Cl^-^ from passing through to anode, thus avoiding the undesired ClOR, while the difference of chemical potentials between the cathode and anode electrolytes can be harvested to reduce the hydrogen production energy cost. The issue related to the formation of Mg^2+^ and Ca^2+^ precipitates during seawater electrolysis is also greatly alleviated in this system. The superhydrophilic Pt_SA_-Ni_6.6_Fe_0.4_P_3_ catalysts on porous Ni foam with lower adhesive force to the bubbles results in fast bubble release from the electrode surface, thus accelerating the mass transport. In situ Raman spectroscopy and DFT calculation reveals that water dissociation is promoted on Pt_SA_-Ni_6.6_Fe_0.4_P_3_ (energy barrier decreases from 0.72 eV on Pt to 0.46 eV), thus reducing the electrochemical polarization for HER in neutral media. In this way, the asymmetric electrolyzer exhibits the current densities of 10 mA cm^−2^ and 100 mA cm^−2^ at low voltages of 1.31 and 1.46 V for direct seawater electrolysis, respectively. It also reaches a current density of 400 mA cm^−2^ at low voltage of 1.66 V at 80 °C, corresponding to the electricity price of US$1.36 per kg of H_2_, which is lower than the DOE 2025 target of US$1.4 per kg of H_2_. The total hydrogen levelized cost is also estimated to be €3.79 per kg of H_2_ according to the calculation framework proposed by Enapter, which is much lower than the projected price for the mass-produced EL Model T in 2023/2024 of €4.15 per kg of H_2_. This work provides an efficient electrolyzer architecture and catalyst design route for direct seawater electrolysis with low energy cost.

## Methods

### Materials

Ammonium chloride (NH_4_Cl, 99.5%), nickel sulfate hexahydrate (NiSO_4_·6H_2_O, 99%), iron sulfate heptahydrate (FeSO_4_·7H_2_O, 99%), diethylene glycol (C_4_H_10_O_3_, 99%), sodium hydroxide (NaOH, ≥99%), ethanol (CH_3_CH_2_OH, ≥99.5%) and sodium chloride (NaCl, ≥99%) were purchased from Innochem. Ni foam (KunShan-Yierda, China, thickness 0.15 cm), phosphorus (Aladdin, >98.9%, −100 mesh), chloroplatinic acid hexahydrate (Aladdin), seawater (from the Yellow sea, China), ruthenium dioxide (RuO_2_, Innochem), commercial Pt/C (Johnson Matthey, 20 wt%) and Nafion® 211 membrane were used as received. Deionized (DI) water (resistivity: 18.3 MΩ cm) was used for the preparation of all aqueous solutions.

### Catalyst syntheses

#### Synthesis of Ni-Fe alloys

Ni-Fe/Ni foam anode was prepared by cathodic electrodeposition of porous Ni microsphere arrays on a pre-shaped Ni foam (0.5 × 1 cm^2^). Typically, the deposition was performed in a standard two electrode configuration at room temperature with an electrolyte of 2.0 M NH_4_Cl, 0.095 M NiSO_4_ and 0.005 M FeSO_4_. Pre-shaped commercial Ni foam and platinum foil were used as the working electrode and counter electrode, respectively. The electrodeposition was carried out at a constant current of −1.5 A cm^−2^ for 5 min to obtain Ni-Fe/NF. Ni-Fe alloys with different NiFe ratios can be obtained by regulating the ratio of NiSO_4_ and FeSO_4_ in the electrolyte.

#### Synthesis of Ni_6.6_Fe_0.4_P_3_ NWs

The Ni_6.6_Fe_0.4_P_3_ NWs were synthesized by a simple solvothermal method. Typically, 600 mg phosphorus was dispersed in 10 mL diethylene glycol and stirred for 30 min. Subsequently, the solution was transferred into 50 mL Teflon-lined autoclave and mixed with the pretreated Ni-Fe foam, maintained at 250 °C for 6 h, later naturally cooled to room temperature. Then, the as-obtained Ni_6.6_Fe_0.4_P_3_ NWs underwent an ethanol-water-ethanol cleaning process for 10 min, respectively.

#### Synthesis of Pt_SA_-Ni_6.6_Fe_0.4_P_3_ NWs

Pt_SA_-Ni_6.6_Fe_0.4_P_3_ was fabricated by the electrochemical reduction process in the three-electrode system, in which the fabricated Ni_6.6_Fe_0.4_P_3_ was performed as the working electrode, graphite sheet acted as a counter electrode, saturated calomel electrode acted as a reference electrode. The corresponding electrochemical process was carried out by multi-cycle cathode polarization in 1 M NaOH solution (100 ml) containing 4 μM H_2_PtCl_6_ with a scan rate of 50 mV s^−1^ between 0 and −0.40 V versus RHE for 800 cycles.

#### Synthesis of Pt_SA_-Ni-Fe

Pt_SA_-Ni-Fe was fabricated by the electrochemical reduction process in the three-electrode system, in which the fabricated Ni-Fe alloy was performed as the working electrode, graphite sheet acted as a counter electrode, saturated calomel electrode acted as a reference electrode. The corresponding electrochemical process was carried out by multi-cycle cathode polarization in 1 M NaOH solution (100 ml) containing 4 μM H_2_PtCl_6_ with a scan rate of 50 mV s^−1^ between 0 and −0.40 V versus RHE for 800 cycles.

#### Synthesis of 20 wt% PtC/NF

The performance of commercial Pt/C were tested after it is supported on Ni foam in this work to keep the close surface area. The ink of 20 wt% commercial Pt/C was covered on Ni foam and the total Pt loading was 60 μg on the Ni foam (0.5 × 1 cm^2^).

#### Preparation of Na^+^ ions exchange membrane

The Na^+^ ions exchange membrane was obtained by the treatment of commercial Nafion® 211 membrane. The commercial Nafion® 211 membrane was first immersed in 5 vol% H_2_O_2_ at 70 °C for 1 h. After cleaning with deionized water, the membrane was then immersed in 1 M NaOH at 70 °C for 12 h to obtain the Na^+^ ions exchange membrane.

#### Assembly of catalysts in asymmetric electrolyzer

The catalysts used in asymmetric electrolyzer was the same as that in half-cell with a size of 0.5 × 1 cm^2^. the Pt loading was about 40 μg (determined by ICP-MS) in the Pt_SA_-Ni_6.6_Fe_0.4_P_3_. Porous catalysts were extruded on the Na^+^ ions exchange membrane carefully and applied in asymmetric electrolyzer.

### Characterizations

The morphology measurement of the synthesized catalysts was performed by SEM (Nova NanoSEM 450 (FEI, USA)). TEM characterizations were obtained on a Tecnai G2 20 (FEI, USA). HRTEM images, HAADF-STEM images, and STEM-EDS mapping images were obtained by a FEI Tecnai F20. The Pt contents in the catalysts were measured by inductively coupled plasma optical emission spectrometry. XRD patterns were collected from Rigaku MiniFlex 600 diffractometer with a Cu radiation source (*λ* = 0.15406 nm. XPS spectra were collected from Thermo Scientific K-Alpha. X-Ray Fluorescence (XRF) results were obtained from M4 TORNADO. Raman measurements were performed using a LabRAM HR Evolution (Horiba JobinYvon, France) with 532 nm excitation wavelength. Extended X-ray absorption fine structure spectroscopy (EXAFS) at the Pt L3-edge was performed at BL11B of Shanghai Synchrotron Radiation Facility.

### Electrochemical measurements

The OER and HER polarization curves were carried out with a CHI 760D (Chenhua, China) electrochemical workstation with a typical three-electrode system at 25 °C, in which fabricated catalysts were directly employed as the working electrode, graphite sheet acted as a counter electrode, saturated calomel electrode (SCE) acted as a reference electrode. The potential was calibrated to reversible hydrogen electrode (RHE) through measuring the potential difference between the SCE and RHE. LSV with 90% iR-compensation were tested under the scan rate of 5 mV s^−1^. The value of iR-compensation was automatically 90% compensated by the CHI 760D. The performance of asymmetric and symmetric electrolyzers were tested on a LAND C3001B battery measurement system (Wuhan, China), and the Chronopotentiometry curves at 10 mA cm^−2^ and 100 mA cm^−2^ were presented with manual 90% iR-compensation. The value of resistance was tested by CHI 760D which was 0.5 ± 0.05 Ω. The area of electrodes in the electrolyzer are 0.5 cm^2^. The flow rate of peristaltic pump is 6 ml min^−1^. The test temperature was controlled by heating the input electrolyte to the specified temperature. The electrochemical active surface area (ECSA) can be estimated with the use of the double-layer capacitances (*C*_dl_). The specific capacitance for a flat surface (*C*_s_) is supposed to be ~ 40 μF cm^−2^, and the ECSA is estimated by the following formula^55^:

$${{{{{\rm{ECSA}}}}}}=\,\frac{{C}_{{{{{{{\rm{dl}}}}}}}}}{{C_{{{{{\rm{s}}}}}}}}$$

### XAFS measurements and data processing

Pt L3-edge XAFS measurements were performed at BL11B station in Shanghai Synchrotron Radiation Facility (SSRF). The electron storage ring of SSRF was operated at 3.5 GeV, with a maximum current of 250 mA. XAFS data were collected using a fixed-exit Si(111) double-crystal monochromator, and the energy was calibrated using metals foil. The samples were pelletized as disks of 13 mm diameter with 1 mm thickness by using LiF power as binder. Utilizing the ATHENA module of the IFEFFIT software packages, the obtained EXAFS data were performed according to the standard procedures^56^ (Nucl. Sci. Tech., 2015, 26, 50102). The EXAFS contributions were separated from different coordination shells by using a hanning windows (dk = 1.0 Å^−1^). Fits were carried out using a k range of 3–13.5 Å^−1^ and a R range of 1.1–2.9 Å using the module ARTEMIS of IFEFFIT. the overall amplitude reduction factor S_0_^2^ was fixed to the best-fit value of 0.81 determined from fitting the data of metal Pt foil.

### In situ Raman experimental setup

The electrochemical Raman measurements were carried out on LabRAM HR800. A Nd-YAG laser with 532 nm excitation wavelength and a 50× microscope objective with a numerical aperture of 0.5 were used in all measurements. Raman frequency was calibrated by a Si wafer during each experiment. In situ electrochemical Raman experiments were employed in a C031 in situ Raman cell from Gaoss Union and a CHI 760D (Chenhua, China) electrochemical workstation was used to control the potential. Each spectrum was obtained with the exposure time of 60 s and accumulating twice.

### DFT calculation details

Theoretical calculations: The Vienna Ab Initio Package (VASP) was employed to perform all the density functional theory (DFT) calculations within the generalized gradient approximation (GGA) using the Perdew, Burke, and Enzerhof (PBE) formulation^57–59^. The projected augmented wave (PAW) potentials were applied to describe the ionic cores and take valence electrons into account using a plane wave basis set with a kinetic energy cutoff of 450 eV^60^. Partial occupancies of the Kohn–Sham orbitals were allowed using the Gaussian smearing method and a width of 0.05 eV. The electronic energy was considered self-consistent when the energy change was smaller than 10^−6^ eV. A geometry optimization was considered convergent when the force change was smaller than 0.03 eV/Å. Grimme’s DFT-D3 methodology was used to describe the dispersion interactions^61^. For implicit solvation calculations, we used VASPsol^54^, a software package that incorporates solvation into VASP within a self-consistent continuum model. VASPsol, due to its simplicity and low computational costs. The energy from DFT is added to the energies from electrostatic interactions between the solute and the solvent and the cavitation energy to create the solute within the solvent. The vacuum spacing perpendicular to the plane of the structure is 20 Å. The Brillouin zone integral utilized the surfaces structures of 2 × 2 × 1 Monkhorst–Pack K-point sampling. The free energy was calculated using the equation:

$$G={E}_{{{{{{\rm{ads}}}}}}}+{{{{{\rm{ZPE}}}}}}-{{{{{\rm{TS}}}}}}$$

where *G*, *E*_ads_, ZPE, and TS are the free energy, total energy from DFT calculations, zero-point energy and entropic contributions, respectively.

## Supplementary information

Supplementary information

Peer Review File

## Data Availability

The experimental data generated in this study have been provided in the Supplementary information. Any additional data are available from the corresponding author.
