# Supplementary material for: A sodium-ion-conducted asymmetric electrolyzer to lower the operation voltage for direct seawater electrolysis
Source: Nat Commun. 2023 Jul 4;14:3934. doi: 10.1038/s41467-023-39681-1 (PMC10319863; doi:10.1038/s41467-023-39681-1)
Supplement: Supplementary file 1 — Supplementary information [file 41467_2023_39681_MOESM1_ESM.pdf]

Supplementary information

A sodium-ion-conducted asymmetric electrolyzer to lower the operation voltage for direct seawater electrolysis

Hao Shi[1], Tanyuan Wang[1]*, Jianyun Liu[1], Weiwei Chen[2], Shenzhou Li[1], Jiashun Liang[1], Shuxia Liu[1], Xuan Liu[1], Zhao Cai[3], Chao Wang[4], Dong Su[2], Yunhui Huang[1], Lior Elbaz[5], Qing Li[1]*

[1] State Key Laboratory of Material Processing and Die & Mould Technology, School of Materials Science and Engineering, Huazhong University of Science and Technology, Wuhan, Hubei 430074, China.

[2] Beijing National Laboratory for Condensed Matter Physics, Institute of Physics, Chinese Academy of Sciences, Beijing, 100190, China.

[3] Faculty of Materials Science and Chemistry, China University of Geosciences (Wuhan), Wuhan, Hubei 430074, China.

[4] School of Materials Science and Engineering, Tongji University, Shanghai 201804, China.

[5] Department of Chemistry and the Institute of Nanotechnology and Advanced Materials, Bar-Ilan University, Ramat-Gan, 5290002, Israel.

Email: wangty@hust.edu.cn (Tanyuan Wang), qing_li@hust.edu.cn (Qing Li)

Supplementary Figures

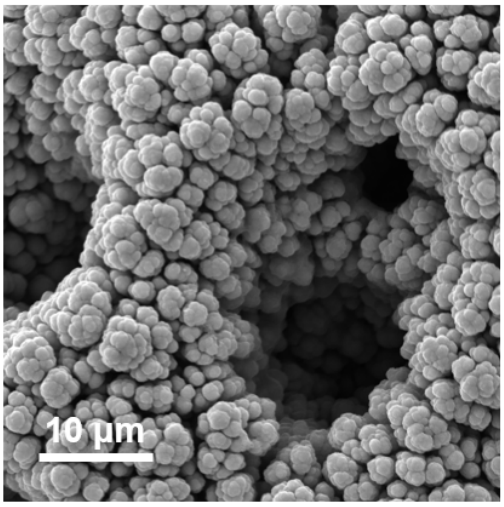

Supplementary Figure 1. The SEM image of hierarchically porous Ni-Fe alloy on Ni foam.

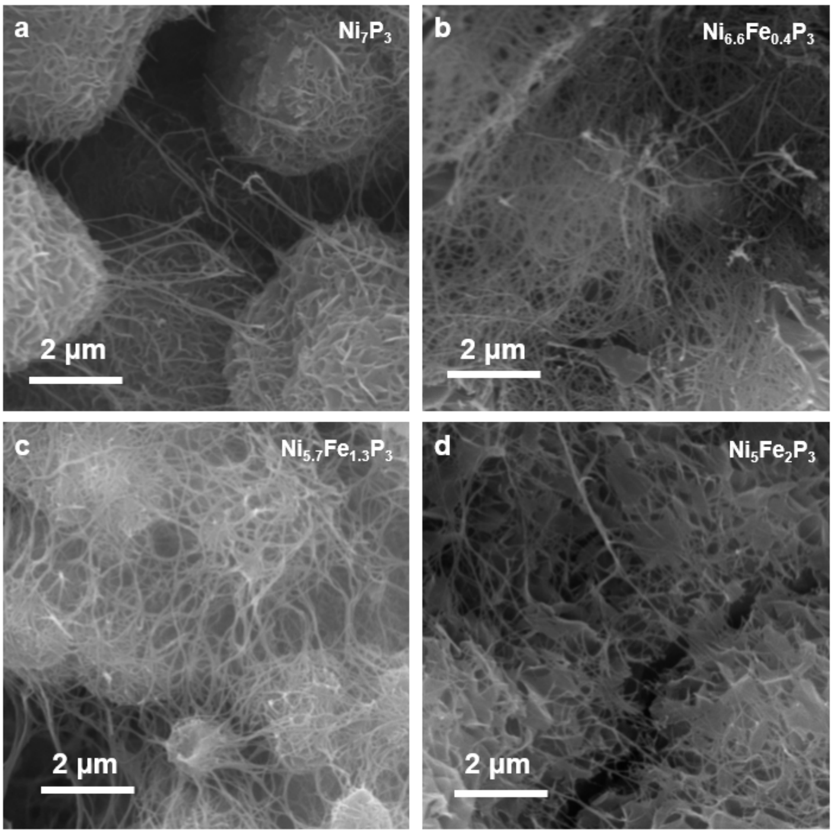

Supplementary Figure 2. The SEM images. (a) Ni_7P_3, (b) $Ni_{6.6}Fe_{0.4}P_3$, (c) $Ni_{5.7}Fe_{1.3}P_3$, (d) $Ni_5Fe_2P_3$.

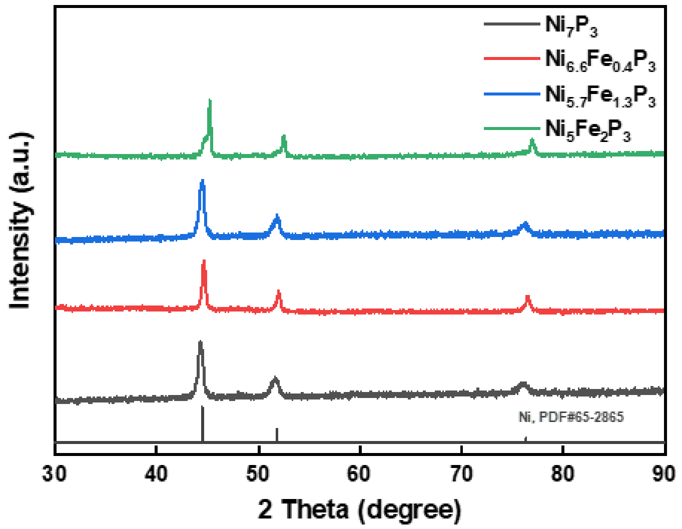

Supplementary Figure 3. The XRD patterns of Ni_7P_3, $Ni_{6.6}Fe_{0.4}P_3$, $Ni_{5.7}Fe_{1.3}P_3$ and $Ni_5Fe_2P_3$.

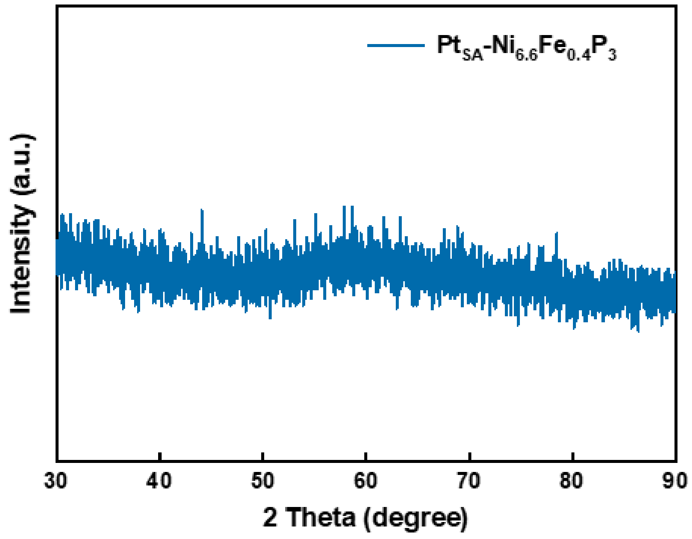

Supplementary Figure 4. The XRD pattern of the powder slightly scraped from the surface of Pt_{SA}-$Ni_{6.6}Fe_{0.4}P_3$.

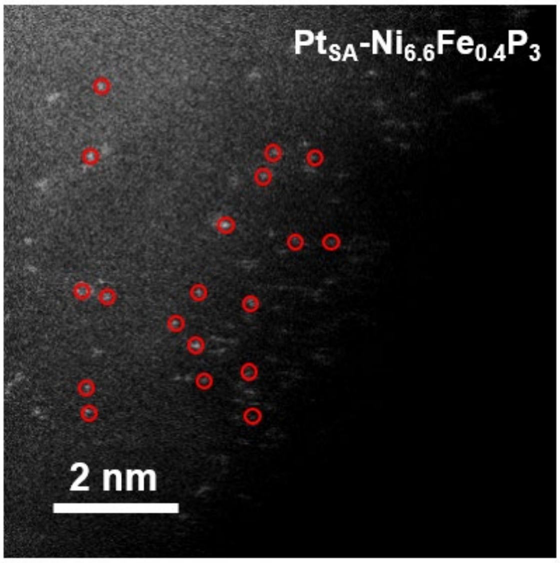

Supplementary Figure 5. The HAADF-STEM images of Pt$_{SA}$-Ni$_{6.6}$Fe$_{0.4}$P$_3$. The part marked with red circles are Pt single atoms.

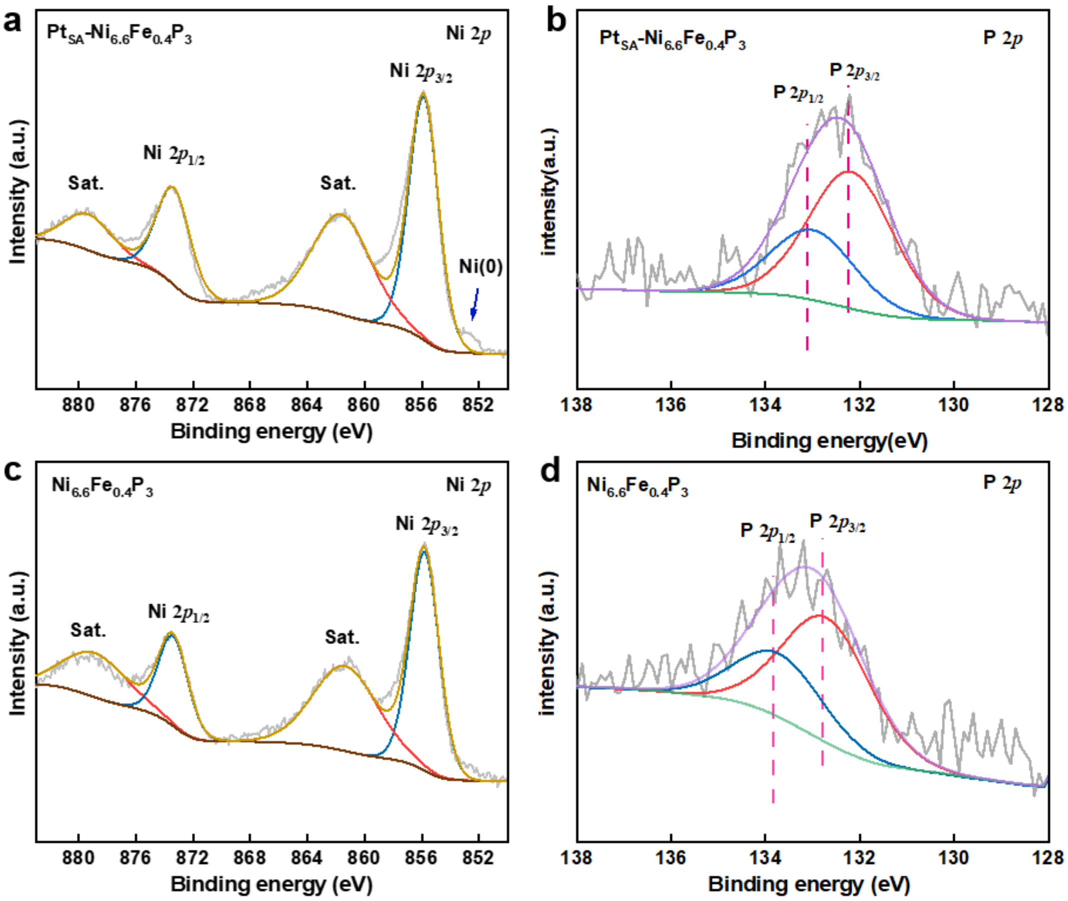

Supplementary Figure 6. The XPS spectrums. (a) Ni 2p of Pt$_{SA}$-Ni$_{6.6}$Fe$_{0.4}$P$_3$. (b) P 2p of Pt$_{SA}$-Ni$_{6.6}$Fe$_{0.4}$P$_3$. (c) Ni 2p of Ni$_{6.6}$Fe$_{0.4}$P$_3$. (d) P 2p of Ni$_{6.6}$Fe$_{0.4}$P$_3$.

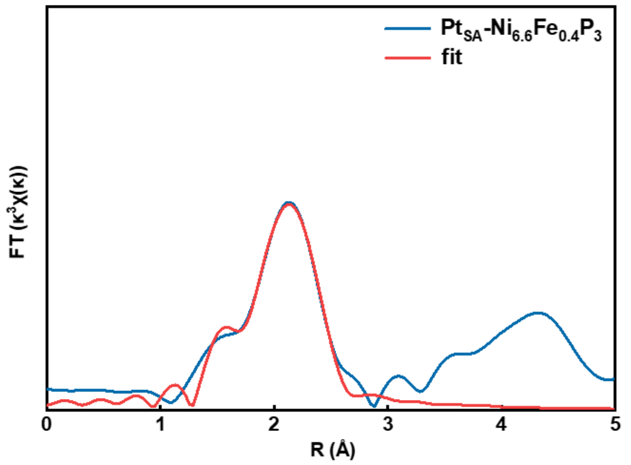

Supplementary Figure 7. EXAFS fitting curve of the first-shell of Pt_{SA}-$Ni_{6.6}Fe_{0.4}P_3$ R-space.

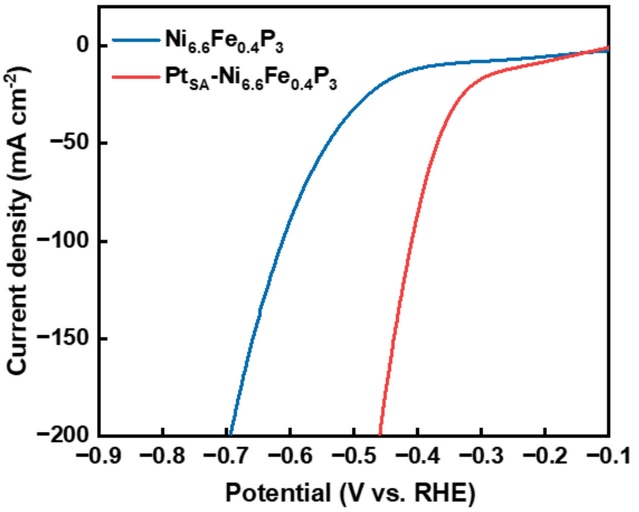

Supplementary Figure 8. HER polarization curves of $Ni_{6.6}Fe_{0.4}P_3$ in 4 M NaCl with 90% iR-compensation (resistance value: 1.1±0.1 Ω).

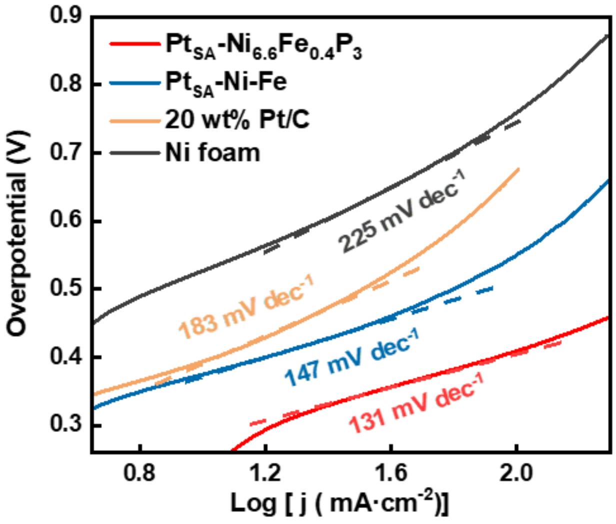

Supplementary Figure 9. Tafel slopes plot of Pt_{SA}-$Ni_{6.6}Fe_{0.4}P_3$, PtSA-Ni-Fe, Pt/C and Ni foam for HER.

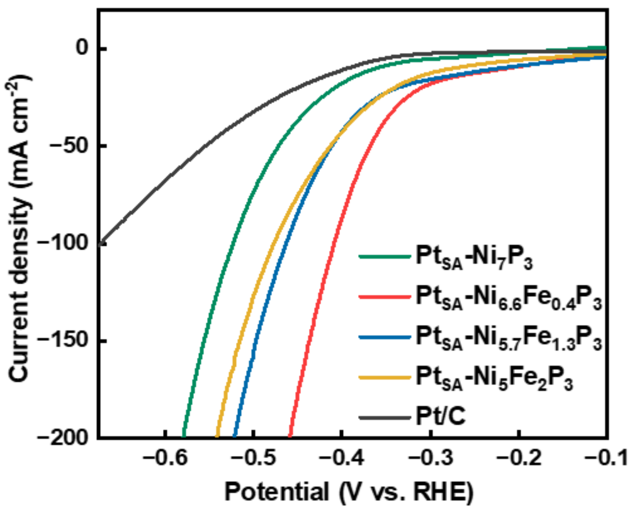

Supplementary Figure 10. HER polarization curves of Pt_{SA}-Ni_7P_3, Pt_{SA}-$Ni_{6.6}Fe_{0.4}P_3$, Pt_{SA}-$Ni_{5.7}Fe_{1.3}P_3$ and Pt_{SA}-$Ni_5Fe_2P_3$ in 4 M NaCl with 90% iR-compensation (resistance value: 1.1±0.1 Ω).

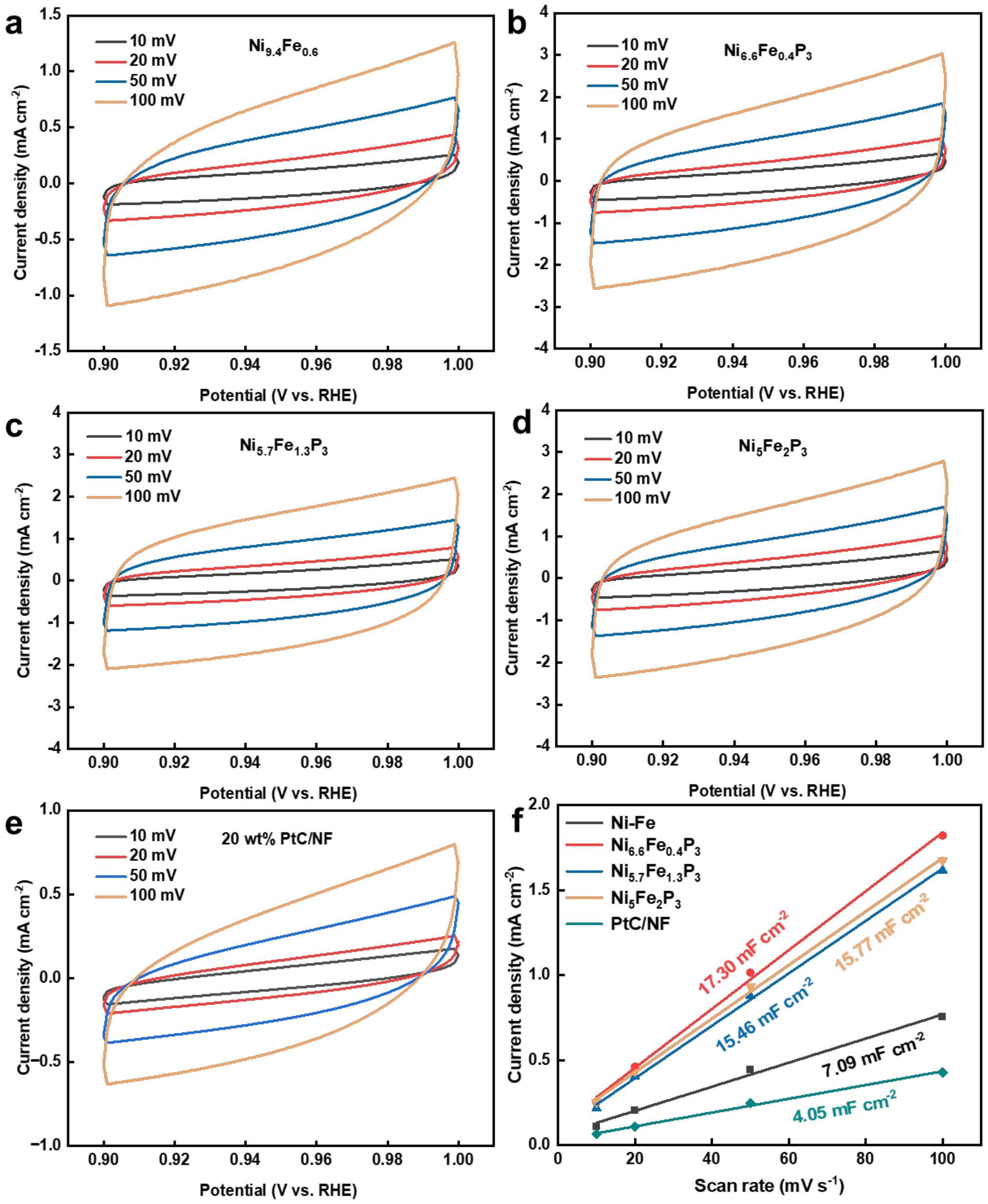

Supplementary Figure 11. Cyclic voltammograms performed in 1 M NaCl in the potential window without the participation of faradaic processes. (a) Ni-Fe alloy, (b) $Ni_{6.6}Fe_{0.4}P_3$, (c) $Ni_{5.7}Fe_{1.3}P_3$, (d) $Ni_5Fe_2P_3$. (e) 20 wt% PtC/NF, (f) The corresponding scan rate dependence of the average currents.

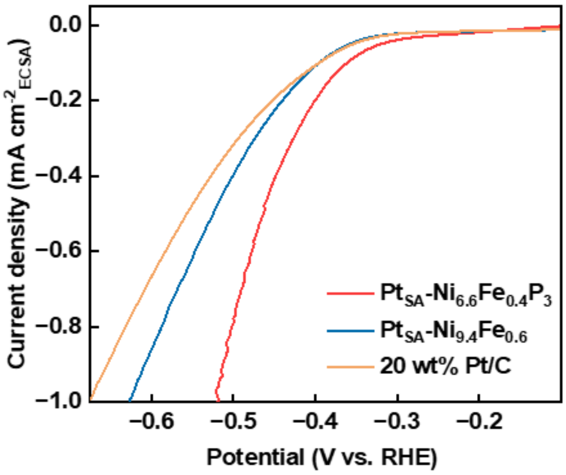

Supplementary Figure 12. The HER polarization curves in 4 M NaCl for different catalysts normalized by ECSA.

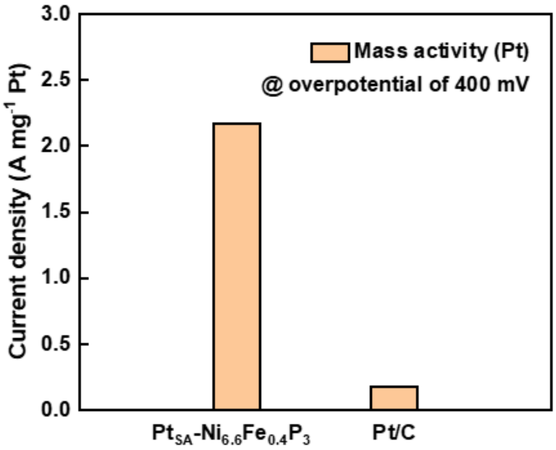

Supplementary Figure 13. The mass activity of Pt_{SA}-$Ni_{6.6}Fe_{0.4}P_3$ and commercial Pt/C at the overpotential of 400 mV.

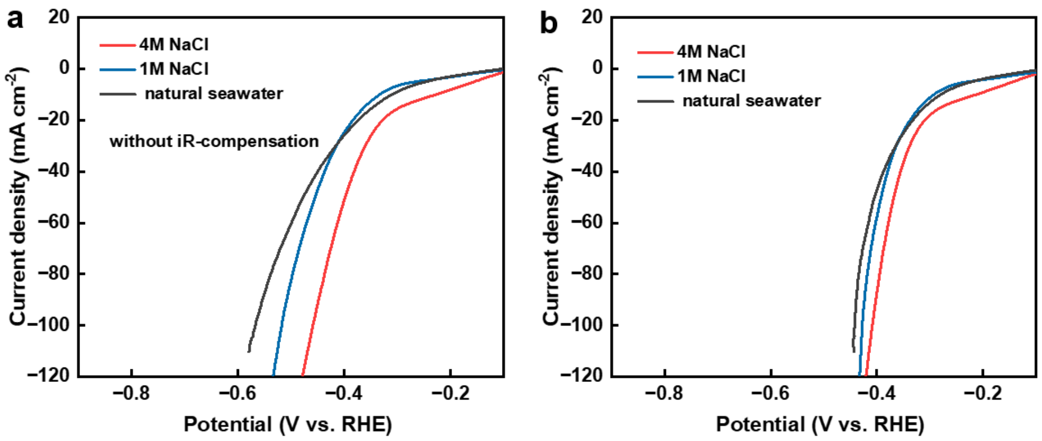

Supplementary Figure 14. HER polarization curves of Pt_{SA}-$Ni_{6.6}Fe_{0.4}P_3$ in 4 M NaCl, 1 M NaCl and natural seawater. (a) without iR-compensation, (b) with 90% iR-compensation (resistance value: 1.1±0.1 Ω for 4 M NaCl, 2.4±0.2 Ω for 1 M NaCl and 3.5±0.2Ω for natural seawater).

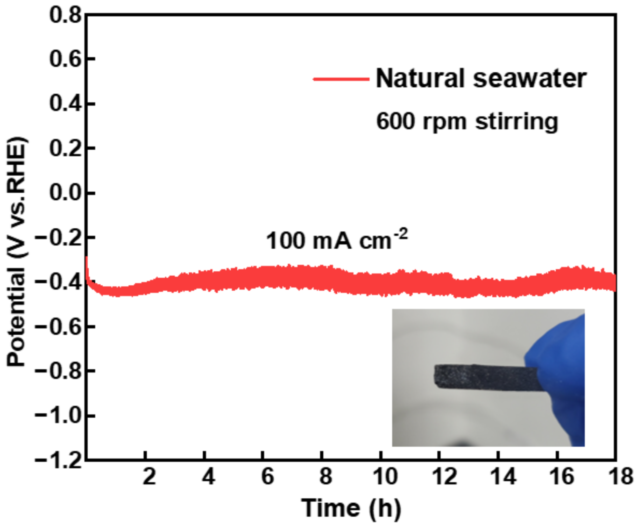

Supplementary Figure 15. The chronopotentiometry curves of Pt_{SA}-$Ni_{6.6}Fe_{0.4}P_3$ at 100 mA cm^{-2} and the photograph of the Pt_{SA}-$Ni_{6.6}Fe_{0.4}P_3$ after stability test in seawater (inset).

White precipitate can hardly be observed on the surface of the catalysts.

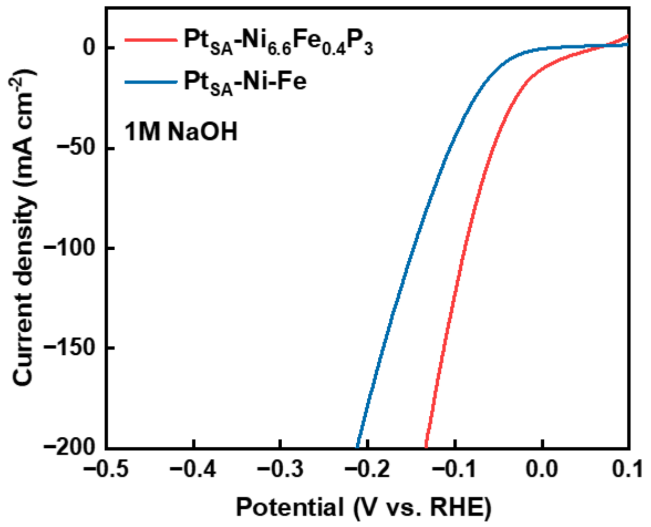

Supplementary Figure 16. HER polarization curves of Pt$_{SA}$-Ni$_{6.6}$Fe$_{0.4}$P$_3$ and Pt$_{SA}$-Ni-Fe in 1 M NaOH with 90% iR-compensation (resistance value: 1.3±0.1 Ω).

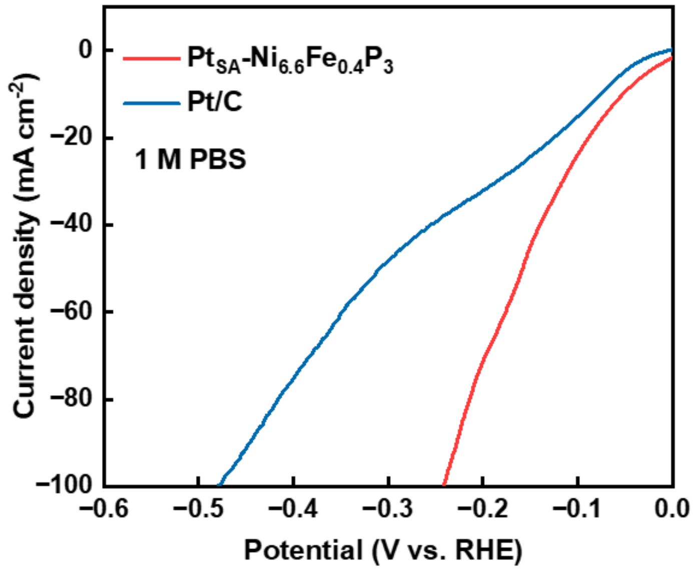

Supplementary Figure 17. HER polarization curves of Pt$_{SA}$-Ni$_{6.6}$Fe$_{0.4}$P$_3$ and Pt/C in 1 M PBS (pH=7) with 90% iR-compensation (resistance value: 2.1±0.2 Ω).

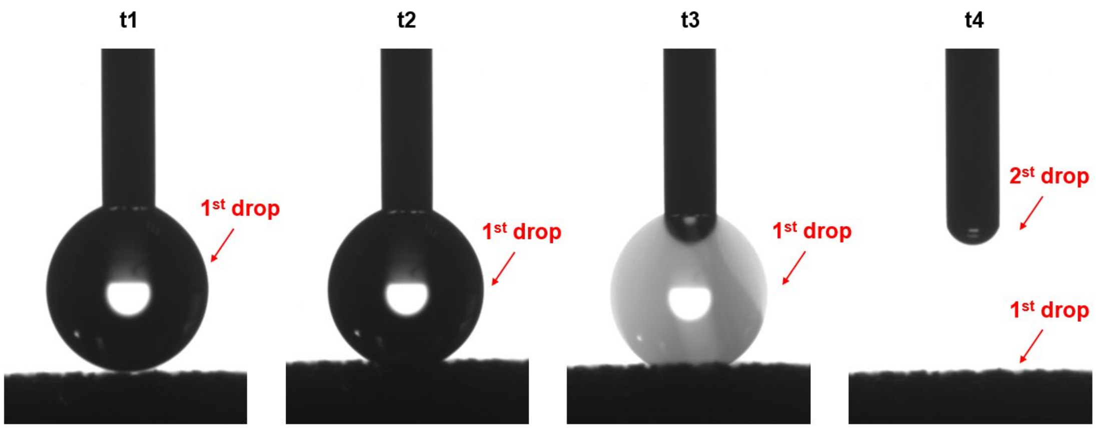

Supplementary Figure 18. Contact angle measurement of Pt_{SA}-$Ni_{6.6}Fe_{0.4}P_3$ electrode.

The wettability of the Ni foam supported catalyst was investigated by measuring the contact angle of the electrode. As shown in Supplementary Figure 17, it was difficult to measure the contact angle of the electrode as the water was absorbed instantaneously, indicating the super hydrophilic nature. The excellent hydrophilicity will boost the electrolyte accessibility, reduce the charge transfer resistance of the electrode[1].

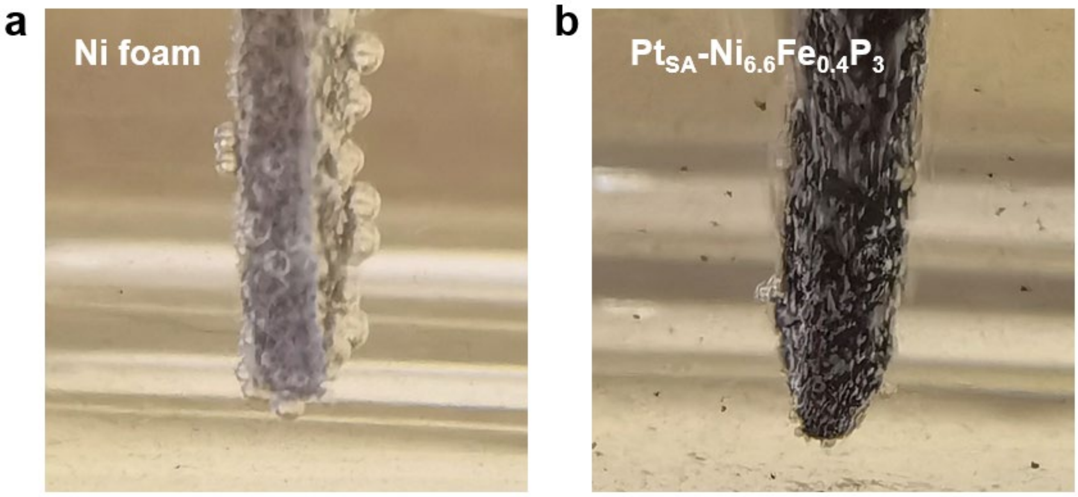

Supplementary Figure 19. The photographs of catalyst during HER process at 100 mA cm^{-2}. (a) Ni foam, (b) Pt_{SA}- $Ni_{6.6}Fe_{0.4}P_3$.

At the same current density, the bubbles on the Ni foam surface are large and sparse,

while Pt_{SA}- $Ni_{6.6}Fe_{0.4}P_3$ electrode surface are small and dense, indicating less adhesion to air bubbles for Pt_{SA}-$Ni_{6.6}Fe_{0.4}P_3$, which facilitates the rapid escape of air bubbles and avoids blockage of the active site.

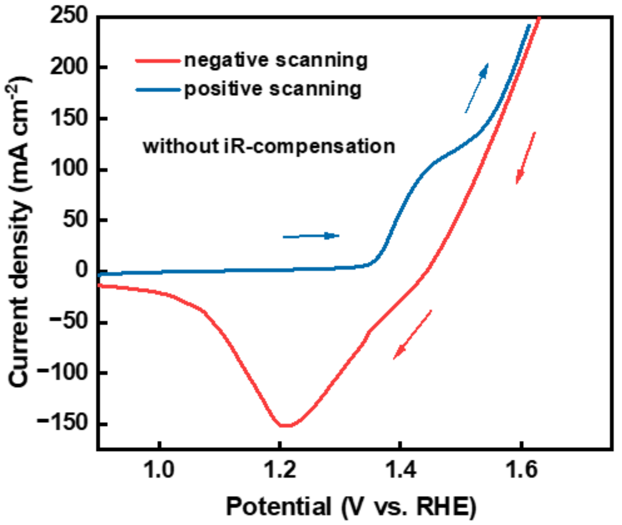

Supplementary Figure 20. OER polarization curves of $Ni_5Fe_2P_3$ in 1 M NaOH by negative scanning and positive scanning without iR-compensation.

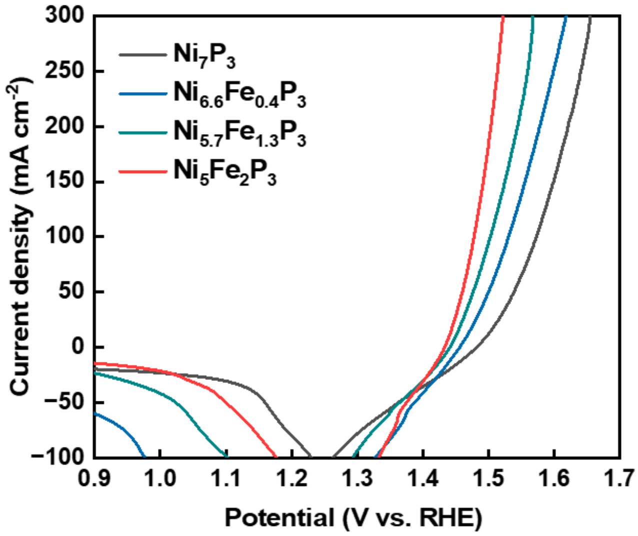

Supplementary Figure 21. OER polarization curves of Ni_7P_3, $Ni_{6.6}Fe_{0.4}P_3$, $Ni_{5.7}Fe_{1.3}P_3$ and $Ni_5Fe_2P_3$ in 1 M NaOH with 90% iR-compensation (resistance value: 1.3±0.1 Ω).

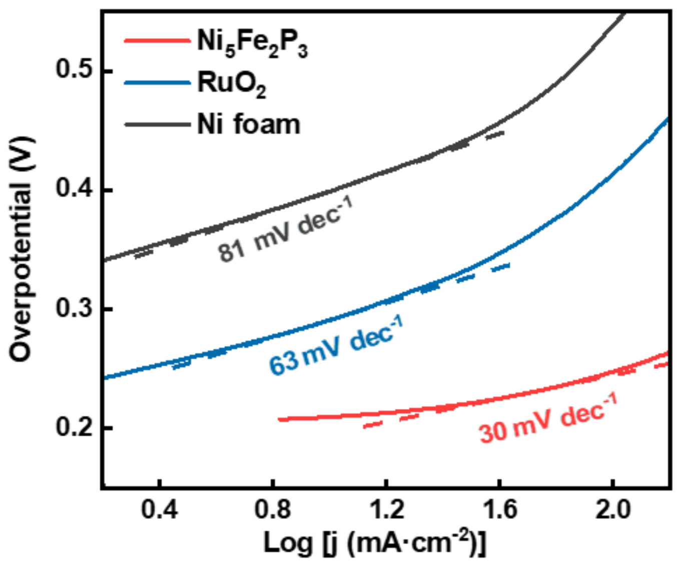

Supplementary Figure 22. Tafel slopes plot of $Ni_5Fe_2P_3$, RuO_2 and Ni foam for OER.

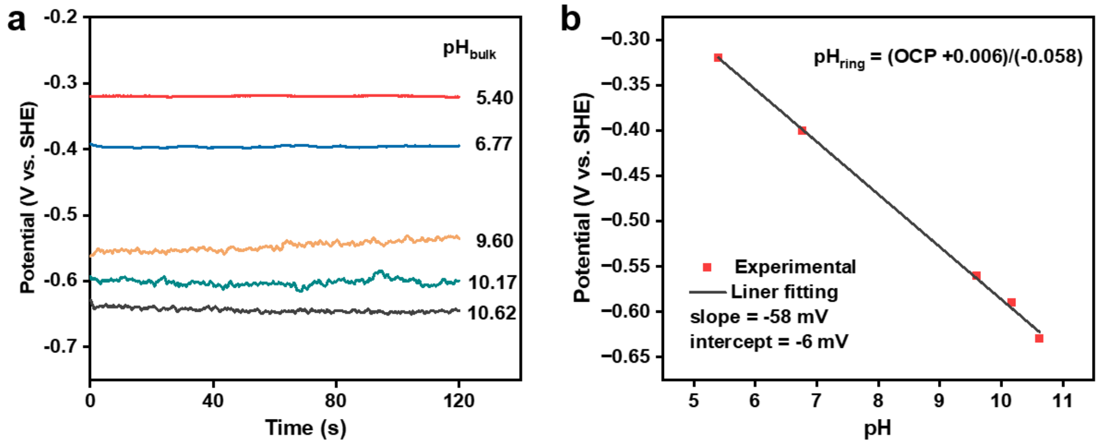

Supplementary Figure 23. (a) Time and (b) pH dependence of open circuit potential (OCP) for ring electrode. The measurement was performed in NaCl solution, and the pH of the bulk electrolyte was changed by adding H_2SO_4 or NaOH.

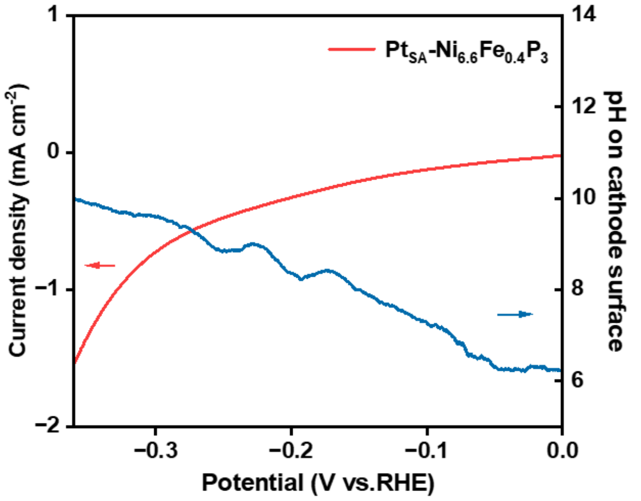

Supplementary Figure 24. The local pH on cathode electrode surface at different potentials (blue line) and the LSV curves (red line).

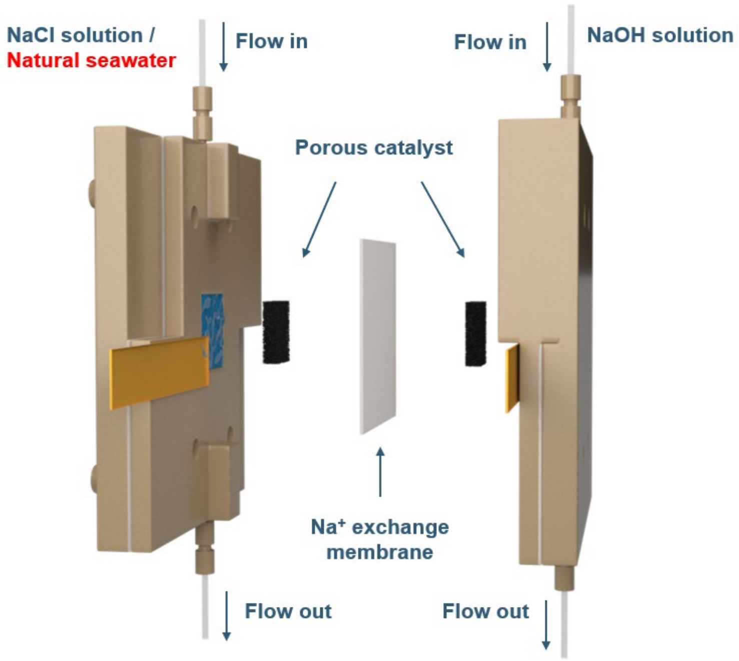

Supplementary Figure 25. The actual assembly scheme of asymmetric electrolyzer with Na^+ exchange membrane.

In this method, the porous superhydrophilic electrodes pressed against the Na^+ ions exchange membrane, and the electrolyte flows across the channels of electrode, while

the cathode and anode electrolyte are in direct contact with the membrane and conductive by Na^+ ions exchange. The catalysts used in asymmetric electrolyzer was the same as that in half-cell with a size of 0.5×1 cm^2. the Pt loading was about 40 μg (determined by ICP-MS) in the Pt_{SA}-$Ni_{6.6}Fe_{0.4}P_3$.

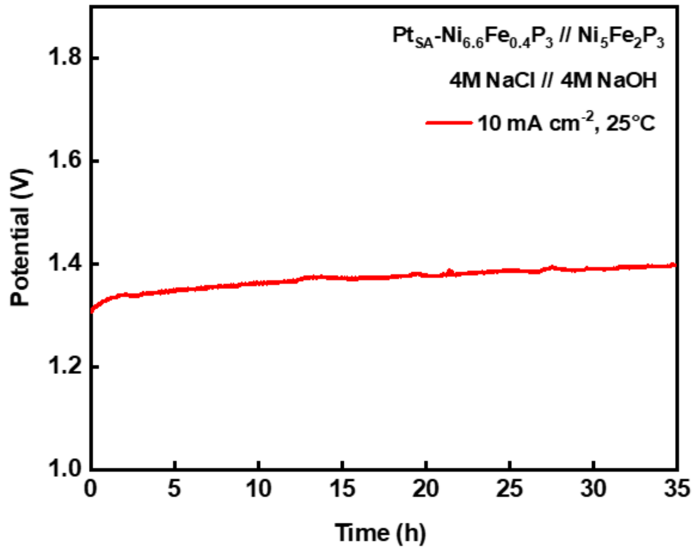

Supplementary Figure 26. Stability test of the asymmetric electrolyzer at constant current density of 10 mA cm^{-2}, 25 °C.

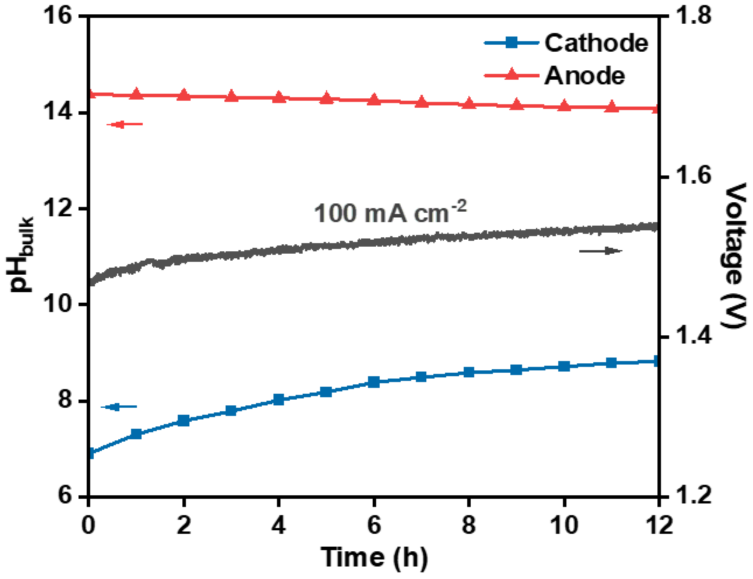

Supplementary Figure 27. The pH of anode and cathode electrolyte of the asymmetric

electrolyzer over time at 100 mA cm^{-2} (electrolyte cycled mode).

After 12 h stability test, the pH of cathode electrolyte increases from 6.9 to 8.8, and the pH of anode electrolyte slightly drops from 14.4 to 14.1, which is consistent with the performance degradation of the asymmetric electrolyzer for direct seawater electrolysis at electrolyte circulation mode.

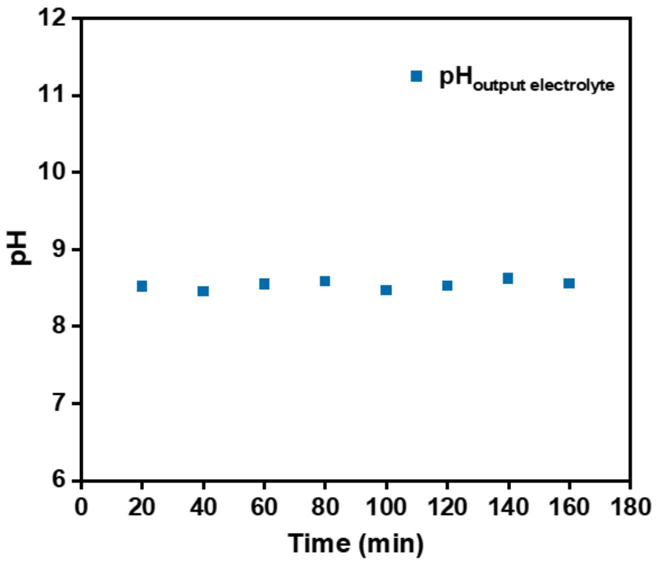

Supplementary Figure 28. The pH of cathode electrolyte with a one-way flowing feeding scheme.

The pH of the output cathode electrolyte could be maintained at about 8.5 in one-way feeding scheme.

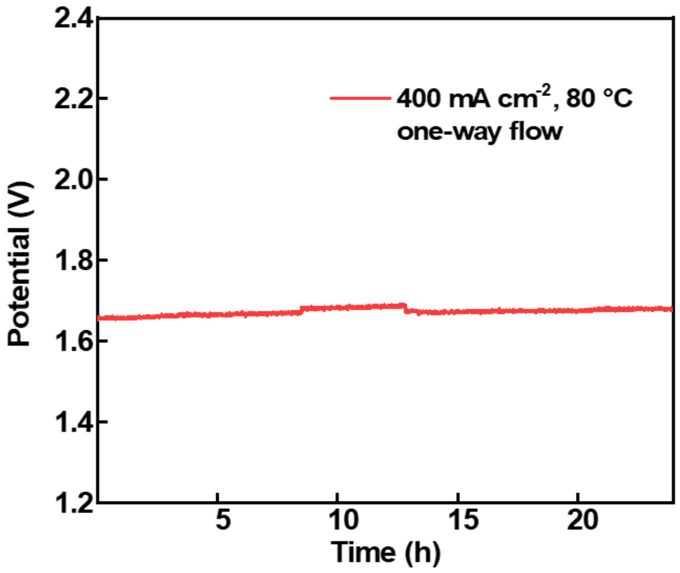

Supplementary Figure 29. The stability test of the asymmetric electrolyzer at a constant current density of 400 mA cm^{-2}, 80 °C with cathode electrolyte one-way feeding scheme (iR-compensation-free).

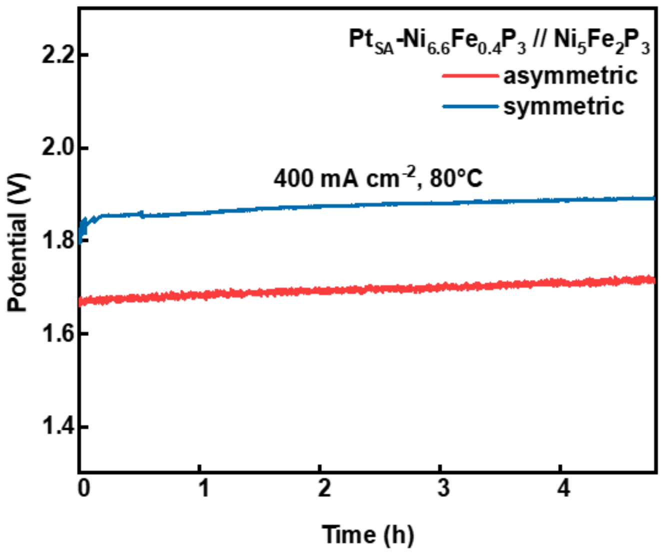

Supplementary Figure 30. The comparison of symmetric and asymmetric electrolyzer at constant current density of 400 mA cm^{-2}, 80 °C without iR-compensation.

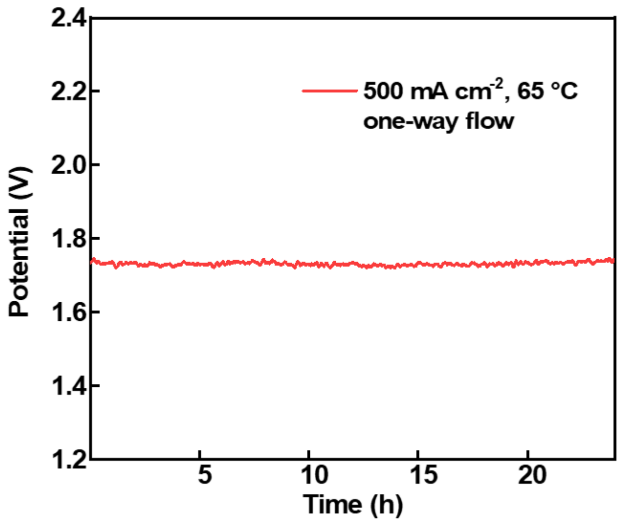

Supplementary Figure 31. The performance of the asymmetric electrolyzer at constant current density of 500 mA cm^{-2}, 65 °C without iR-compensation.

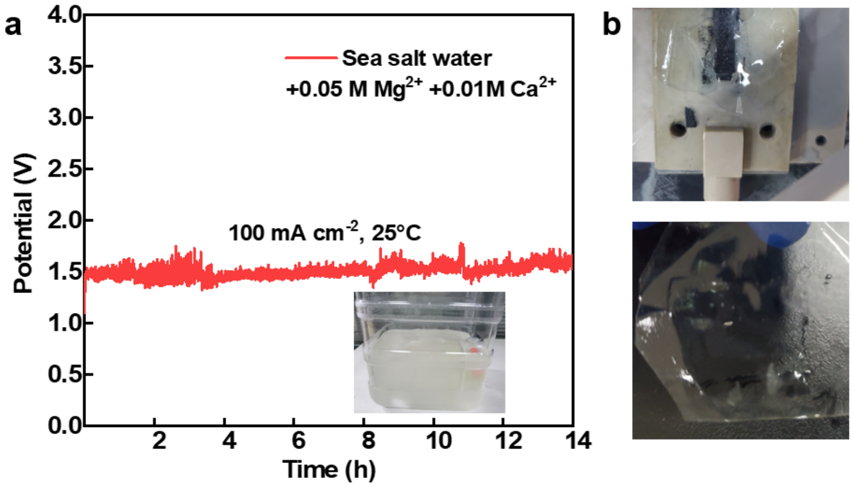

Supplementary Figure 32. The resistance test of the formation of $Mg(OH)_2$ and $Ca(OH)_2$ precipitates for asymmetric electrolyzer in direct sea salt water electrolysis. (a) the stability test in sea salt water with extra Mg^{2+} and Ca^{2+} in a one-way flowing feed scheme and the photo of turbid sea salt water (inset). (b) the photos of the membrane and catalysts after long-term test.

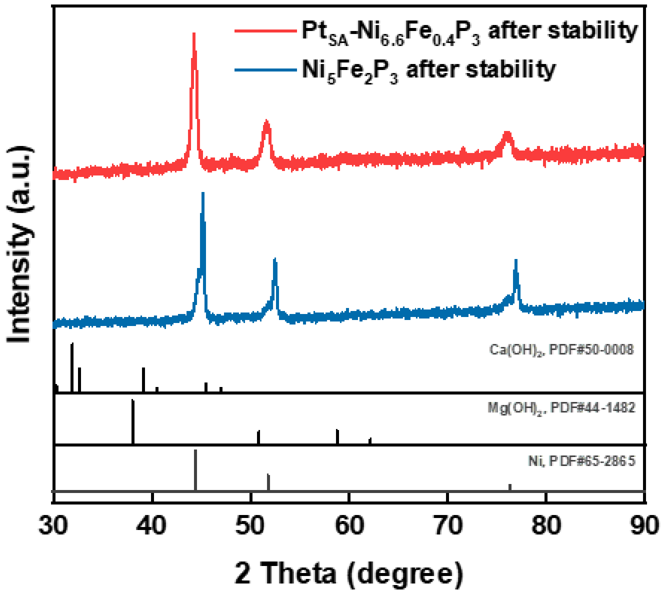

Supplementary Figure 33. The XRD patterns of Pt_{SA}- $Ni_{6.6}Fe_{0.4}P_3$ and $Ni_5Fe_2P_3$ after long-term stability test**.**

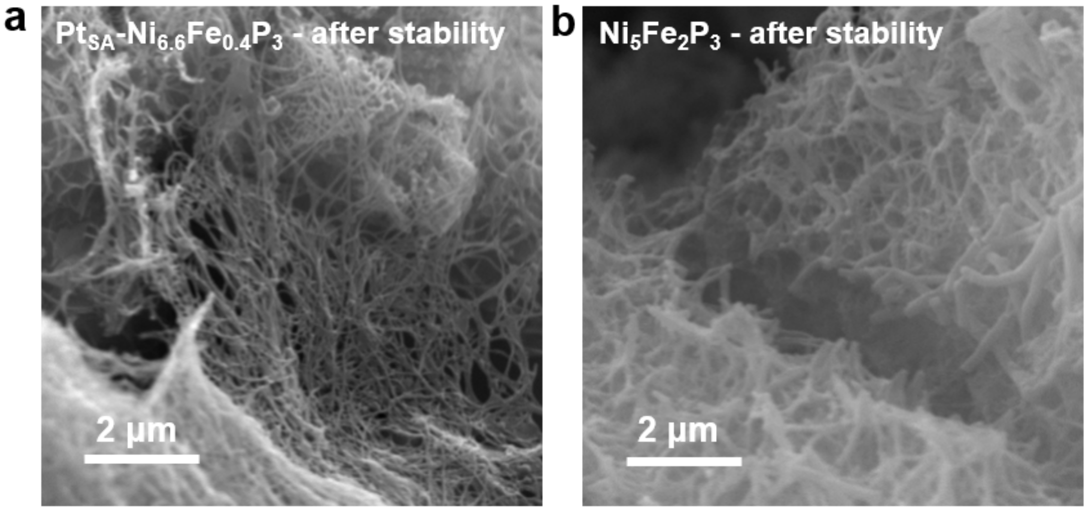

Supplementary Figure 34. The SEM images of catalysts after long-term stability test. (a) Pt_{SA}- $Ni_{6.6}Fe_{0.4}P_3$, (b) $Ni_5Fe_2P_3$.

After long-term stability test, Pt_{SA}- $Ni_{6.6}Fe_{0.4}P_3$ catalyst still maintains the original morphology of nanowires. After a long-time OER test, the $Ni_5Fe_2P_3$ nanowires become

thicker due to the possible in-situ reconstruction

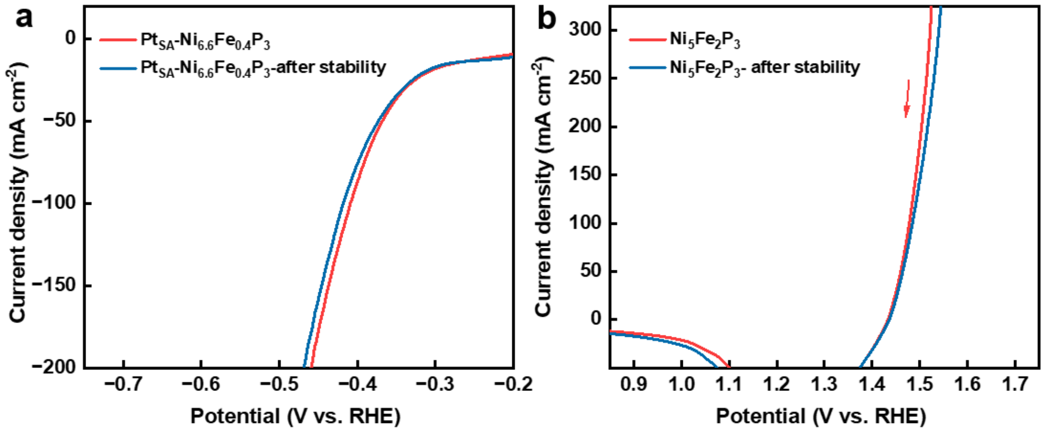

Supplementary Figure 35. Polarization curves after long-term stability test. (a) the HER polarization of Pt_{SA}- $Ni_{6.6}Fe_{0.4}P_3$, (b) the OER polarization of $Ni_5Fe_2P_3$.

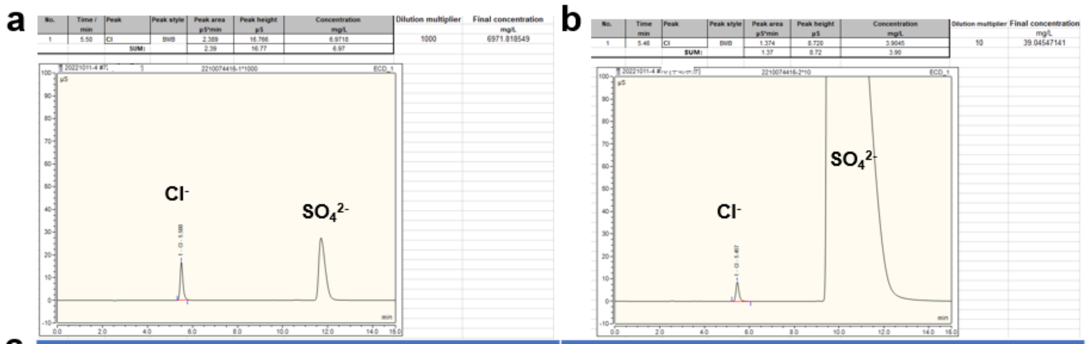

c

Style of membrane	Concentration of Cl^- / M
Anion exchange membrane	0.1964
Na^+ exchange membrane	0.0011

Supplementary Figure 36. The Ion Chromatography test of anode electrolytes after 12 h electrolysis. (a) asymmetric electrolyzer with anion exchange membrane, (b) asymmetric electrolyzer with Na^+ exchange membrane, (c) the corresponding concentration of Cl^- in anode electrolyte. (The electrolytes were adjusted to pH7 by H_2SO_4 before The Ion Chromatography test.)

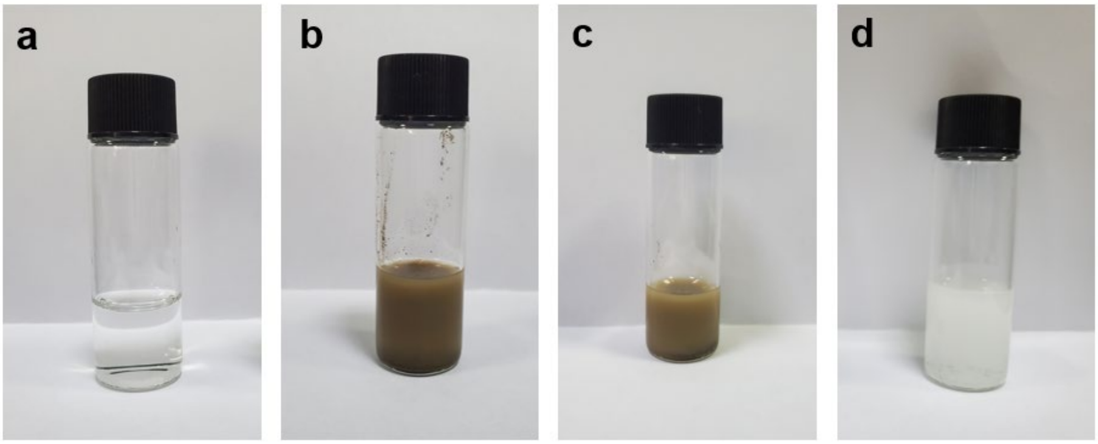

Supplementary Figure 37. The photographs of anode electrolytes. (a) anode electrolyte before electrolysis, (b) anode electrolyte (before electrolysis) after adding $AgNO_3$ solution, (c) anode electrolyte (after electrolysis in asymmetric electrolyzer with Na^+ ions exchange membrane) after adding $AgNO_3$ solution, (d) anode electrolyte (after electrolysis in asymmetric electrolyzer with anion exchange membrane) after adding $AgNO_3$ solution.

When adding $AgNO_3$ solution, a yellowish-brown precipitate appears in the anode electrolyte after electrolysis in asymmetric electrolyzer with Na^+ ions exchange membrane, which is attributed to Ag_2O. Because of the presence of large amounts of Cl^- in the anode electrolyte after electrolysis in asymmetric electrolyzer with anion exchange membrane, when $AgNO_3$ solution was added, a large amount of white AgCl precipitate with much lower solubility appeared in the solution.

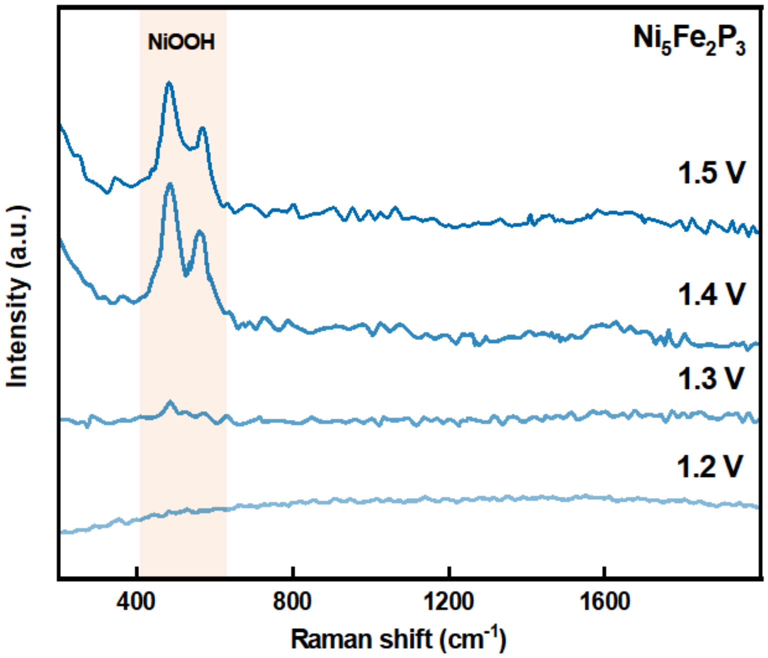

Supplementary Figure 38. *In-situ* Raman spectroscopy of $Ni_5Fe_2P_3$ at OER region (1.2 V_{RHE} to 1.5 V_{RHE}) in 1 M NaOH.

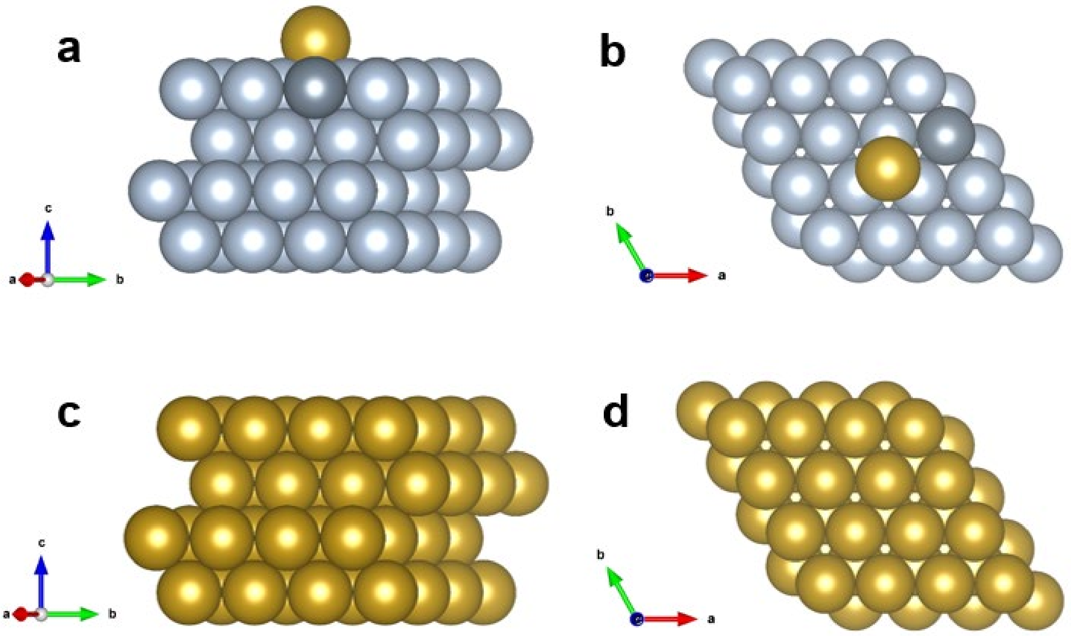

Supplementary Figure 39. The calculation models. (a-b) Pt_{SA}- Ni-Fe along a* and c axis, (c-d) Pt along a* and c axis. (grey: nickel, dark grey: iron, yellow: platinum)

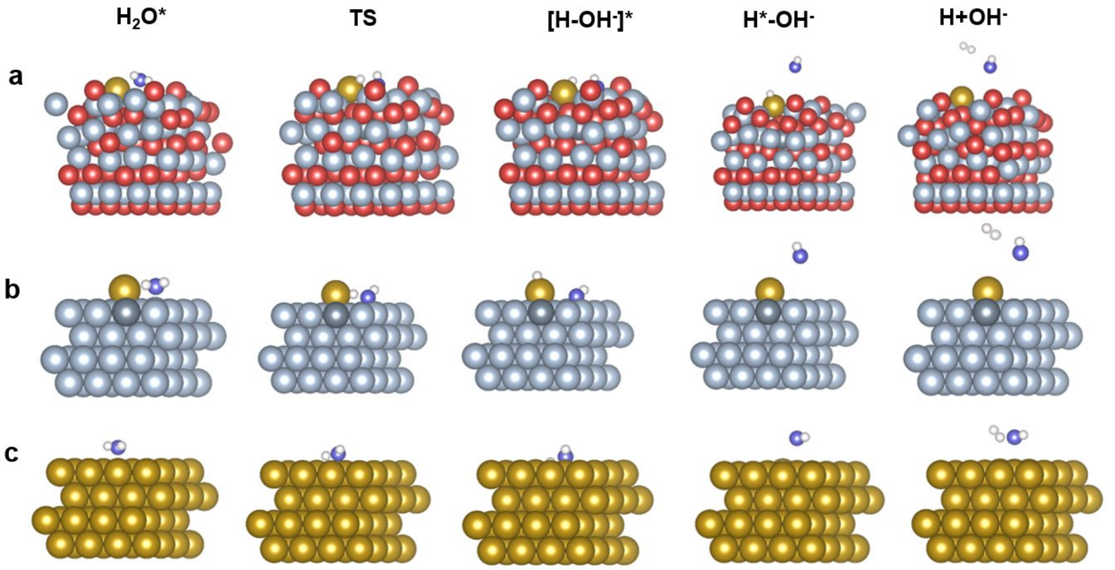

Supplementary Figure 40. Structural configurations of various states for HER in NaCl solution. (a) Pt_{SA}- $Ni_{6.6}Fe_{0.4}P_3$, (b) Pt_{SA}- Ni-Fe, (c) Pt foil. (grey: nickel, dark grey: iron, yellow: platinum, blue: oxygen, white: hydrogen)

Supplementary Table 1. The actual percentages of atoms tested by X-ray Fluorescence Spectrometry.

**catalyst**	**Ni^{2+}/Fe^{2+} of electrodeposition electrolyte**	**Ni at%**	**Fe at%**	**P at%**
$Ni_{6.6}Fe_{0.4}P_3$	9.5/0.5	63.9	4.2	31.9
$Ni_{5.7}Fe_{1.3}P_3$	8/2	56.8	12.8	30.4
$Ni_5Fe_2P_3$	7/3	51.2	19.2	29.6

Supplementary Table 2. FT-EXAFS fitting results of Pt_{SA}-$Ni_{6.6}Fe_{0.4}P_3$ and Pt foil was given as a reference.

	**Shell**	**CN**	**R (Å)**	**σ^2**	**ΔE_0**	**R factor**
**Pt foil**	Pt-Pt	12	2.76±0.01	0.005	4.45±0.3	0.0019
**Pt_{SA}-$Ni_{6.6}Fe_{0.4}P_3$**	Pt-O	0.96±0.6	2.02±0.01	0.004	7.58±2.8	0.016
	Pt-Ni	3.31±0.6	2.55±0.01	0.005		

Supplementary Table 3. The percentages of atoms of catalysts after stability test.

Catalyst	Pt at%	Ni at%	Fe at%	P at%
Pt_{SA}-$Ni_{6.6}Fe_{0.4}P_3$	0.4	66.4	4.0	29.2
$Ni_5Fe_2P_3$	/	51.2	20.3	28.5

Supplementary Notes

Supplementary Note 1. The theoretical voltage of water splitting for asymmetric electrolyzer.

Depending on the pH of the electrolyte, the two half reactions and corresponding electrode potentials are given as below:

HER (cathode): $2H_2O + 2e^- = H_2 + 2OH^-$

$$E_{H^+/H_2O} = E^{\theta}_{H^+/H_2O} + \frac{RT}{2F} ln \frac{[\alpha_{H_2O}]^2}{[\alpha_{H_2}][\alpha_{OH^-}]} \quad (1)$$

$$= 0\ \text{V} - 0.059 \times pH_{\text{cathode}}$$

$$= -0.413\ \text{V}\ (pH = 7)$$

OER (cathode): $4OH^- = 2H_2O + O_2 + 4e^-$

$$E_{O^2/OH^-} = E^{\theta}_{O^2/OH^-} + \frac{RT}{4F} ln \frac{[\alpha_{H_2O}]^2[\alpha_{O_2}]}{[\alpha_{OH^-}]^4} \quad (2)$$

$$= 1.229\ \text{V} - 0.059 \times pH_{\text{anode}}$$

$$= 0.403\ \text{V}\ (pH = 14)$$

The theory cell voltage of the asymmetric electrolyzer for direct water splitting.

$$E_{cell} = E_{H^+/H_2O} - E_{O^2/OH^-} = \text{-0.413 V-0.403 V= -0. 816 V} \quad (3)$$

Supplementary Note 2. The local pH test on the surface of Pt_{SA}-$Ni_{6.6}Fe_{0.4}P_3$.

A rotating ring-disk electrode (RRDE) was used to detect the local pH of the electrode surface. The local pH on the ring electrode is calculated from the open circuit potential (OCP) of the ring electrode since the OCP obeys the Nernst equation under the condition that hydrogen oxidation/evolution reactions (HOR/HER) proceed reversibly.

Measuring pH on the ring electrode do not disturb the reactions on the disk electrode. In addition, the flow from the disk to the ring electrodes can be described accurately by a convective-diffusion equation, hence the local *pH* observed at the ring electrode can be converted into the local pH immediately adjacent to the disk electrode[2].

To confirm that the OCP of the Pt ring electrode accurately reflected the hydrogen equilibrium potential, the ring OCPs are measured with solutions of various pH values without any reactions at the disk electrode. The time dependence of the ring OCP is shown in Supplementary Figure 23a. The pH values shown in the figure are those in the bulk solution measured by a pH meter. We take a linear fit of OCPs response to pH of the ring, in which the slope is 58 mV/pH and the intercept is −6 mV, showing a good agreement with Nernst equation (Supplementary Figure 23).

Next, the measurement of pH on the catalyst surface is performed under different applied potentials. Pt_{SA}-$Ni_{6.6}Fe_{0.4}P_3$ is scraped off the Ni foam and then loaded on the disk electrode. Linear sweep voltammetry is performed on the catalyst-loaded disk electrode in NaCl solution, and meanwhile, OCPs are recorded on the ring electrode. The pH value of the ring electrode is evaluated from the OCPs using the equation.

$$\mathrm{pH_{ring}} = (\mathrm{OCP} + 0.006\,\mathrm{V})/(-0.058\,\mathrm{V}/pH) \quad (4)$$

The pH value of the catalyst-loaded disk electrode can be deducted from the pH value of the ring electrode by the following equation[2]:

$$C_{\mathrm{H^+,ring}} - C_{\mathrm{OH^-,ring}} = \mathrm{N_D} \times (C_{\mathrm{H^+,disk}} - C_{\mathrm{OH^-,disk}}) + (1\text{-}\mathrm{N_D}) \times (C_{\mathrm{H^+,bulk}} - C_{\mathrm{OH^-,bulk}}) \quad (5)$$

where $C_{\mathrm{H^+,ring}}$ and $C_{\mathrm{H^+,disk}}$ are the concentrations of H^+ on the ring and disk electrode, respectively, $C_{\mathrm{OH^-,ring}}$ and $C_{\mathrm{OH^-,disk}}$ are the concentrations of OH^- on the ring and disk electrode, respectively, $C_{\mathrm{H^+,bulk}}$ and $C_{\mathrm{OH^-,bulk}}$ are the concentrations of H^+ and OH^- in the bulk solution, respectively, and $\mathrm{N_D} = 0.38$ is the collection efficiency of the ring electrode.

Supplementary Note 3. Calculation of electricity cost.

Cost (H_2/kg) = energy consumption × electricity bill[3] (6)

= 44 kW h/kg H_2 × \$0.031/kW h

= \$1.36 /kg H_2

According to the DOE target[3], the total hydrogen levelized cost is US\$2 /kg H_2, where the feedstock cost contribution (electricity cost and water cost) is \$1.4 /kg H_2 (the price of electricity is \$0.031 /kWh). The feedstock cost in our work is \$1.36 /kg H_2, which is still lower than that of DOE target. The total hydrogen levelized cost of the asymmetric electrolyzer was estimated by the way of adding the other cost (capital cost contribution, fixed O&M cost contribution and other valuable cost contribution) according to the DOE target. The value is US\$1.96 /kg H_2, which is also slightly lower than the DOE target of US\$2 /kg H_2.

According to the Enapter's solution to price which considered the whole lifetime and Capex of the electrolyzer[4], the Capex of EL Model T in 2023/2024 was assumed as the Capex for the asymmetric electrolyzer (€1.4 /kg H_2). And the Opex (€2.39 /kg H_2) of the electrolyzer consists of the electricity cost (€2.2 /kg H_2, electricity bill of €0.05 / kWh), water cost (€0.01 /kg H_2, the water purification cost could be saved in this system) and maintenance cost (€0.18 /kg H_2). And the whole cost of green hydrogen production of the asymmetric electrolyzer for seawater electrolysis is pre-estimated to be €3.79 /kg H_2, which is lower than the EL Model T (€4.15 /kg H_2) in 2023/2024.

We calculate the power (0.664 W/cm^{-2}) and efficiency (75.5%) of the asymmetric electrolyzer operated at 400 mA cm^{-2}, which is higher than the efficiency of commercial alkaline water electrolysis which range between 63%-71% (Current density: 0.2-0.6 A cm^{-2}), indicating that the asymmetric electrolyzer could decrease energy consumption for seawater electrolysis[5].

The lower heating value (LHV) of H_2 is applied to calculate the efficiencies of Pt_{SA}-$Ni_{6.6}Fe_{0.4}P_3$ in the asymmetric electrolyzers; the rate of O_2 generation at 400 mA cm^{-2} is 6.24×10^{17} O_2 cm^{-2} s^{-1}; the rate of H_2 generation at 400 mA cm^{-2} is 1.25×10^{18} H_2 cm^{-2} s^{-1}; LHV of H_2 is 120 kJ g^{-1} H_2; H_2 power out is 0.501 W cm^{-2}.

Supplementary Note 4. The analysis of *in-situ* Raman spectroscopy of $Ni_5Fe_2P_3$ for OER.

Two new bands at 473 cm^{-1} and 551 cm^{-1} appears when the potential increases to 1.4 V, which are attributed to the e_g bending vibration and the A_{1g} stretching vibration of Ni−O in NiOOH, suggesting that the formation of OER active MOOH species with remained P could be very quickly during OER test[6].

Supplementary Note 5. The models for DFT calculations.

Pt_{SA}-$Ni_{6.6}Fe_{0.4}P_3$ model: In the literature, the long-range disorder structure of amorphous materials is usually known as a structure with abundant defects, and it is too complex to directly model an amorphous structure. To avoid the heavy simulations of the amorphous structure, the defective crystal could be used to approximately represent the amorphous structure for modeling and simulation[7]. The model of Pt_{SA}-$Ni_{6.6}Fe_{0.4}P_3$ is established based on (4×4) NiP (111), in which 3/4 of the atoms in the first layer and 1/4 of the atoms in the second layer are missing to simulate vacancy[8]. Some of the Ni atoms are replaced with Fe atoms in actual proportion. The Pt single atom is put on the surface 3-fold hollow Ni site, which is consistent with the results of EXAFs fitting.

Pt_{SA}-Ni-Fe model: The model of Pt_{SA}-Ni-Fe is established based on (4×4) cubic Ni (111), in which some of the Ni atoms are replaced with Fe atoms in actual proportion. The Pt single atom is put on the surface 3-fold hollow Ni site.

Pt foil model: The model of Pt foil is established based on (4×4) Pt (111).

Supplementary Note 6. The reaction paths for DFT calculation.

Pt_{SA}-$Ni_{6.6}Fe_{0.4}P_3$: H_2O is firstly adsorbed on the Ni site adjacent to Pt. And at the final state, the dissociated H_{ad} and OH_{ad} occupy the exposed Pt site and the 3-fold hollow Ni site, respectively. Subsequently, the produced H* on Pt sites could form H_2[8].

Pt_{SA}-Ni-Fe: water is firstly absorbed on the Ni site adjacent to Pt. And at the final state, the dissociated H_{ad} and OH_{ad} occupy the exposed Pt site and the bridge Ni-Ni sites, respectively, followed by the produced H* on Pt sites combine to H_2[9].

Pt foil: the water firstly absorbed on the Pt atoms on Pt (111). And at the final state, the dissociated H_{ad} and OH_{ad} occupy the 3-fold hollow Pt site and bridge Pt-Pt site, respectively. Subsequently, the produced H* on 3-fold hollow Pt site is desorbed to form H_2[10].

Supplementary References

1 Xu, W., Lu, Z., Sun, X., Jiang, L. & Duan, X. Superwetting Electrodes for Gas-Involving Electrocatalysis. *Acc. Chem. Res.* **51**, 1590-1598 (2018).

2 Yokoyama, Y., Miyazaki, K., Miyahara, Y., Fukutsuka, T. & Abe, T. In Situ Measurement of Local pH at Working Electrodes in Neutral pH Solutions by the Rotating Ring-Disk Electrode Technique. *ChemElectroChem* **6**, 4750-4756 (2019).

3 U.S. Department of Energy. Fuel cell technologies office multi-year research, development, and demonstration plan: 3.1 Hydrogen Productionproduction.

4 Thomas Chrometzka, C. J., Aoibhin Quinn, Tanai Potisat. H2 View Exclusive – Calculating the Cost of Green Hydrogen (2020). https://www.enapter.com/newsroom/h2-view-exclusive-calculating-the-cost-of-green-hydrogen

5 Keçebaş, A., Kayfeci, M. & Bayat, M. in *Solar Hydrogen Production* (eds Francesco Calise *et al.*) 299-317 (Academic Press, 2019).

6 Zhu, K., Zhu, X. & Yang, W. Application of In Situ Techniques for the Characterization of NiFe-Based Oxygen Evolution Reaction (OER) Electrocatalysts. *Angew. Chem. Int. Ed. Engl.* **58**, 1252-1265 (2019).

7 Che, Q., Li, Q., Chen, X., Tan, Y. & Xu, X. Assembling amorphous (Fe-Ni)Co-OH/Ni_3S_2 nanohybrids with S-vacancy and interfacial effects as an ultra-highly efficient electrocatalyst: Inner investigation of mechanism for alkaline water-to-hydrogen/oxygen conversion. *Appl. Catal. B Environ.* **263** (2020).

8 Zhao, W. *et al.* Water Dissociative Adsorption on NiO(111): Energetics and Structure of the Hydroxylated Surface. *ACS Catal* **6**, 7377-7384 (2016).

9 Zhao, L. *et al.* Steering elementary steps towards efficient alkaline hydrogen evolution via size-dependent Ni/NiO nanoscale heterosurfaces. *Natl. Sci. Rev.* **7**, 27-36 (2020).

10 Fajin, J. L., MN, D. S. C. & Gomes, J. R. Density functional theory study of the water dissociation on platinum surfaces: general trends. *J. Phys. Chem. A* **118**, 5832-5840 (2014).
